# Residual-activated smooth sliding-mode control for actuator-limited quadrotor UAV attitude–altitude tracking

**DOI:** 10.1371/journal.pone.0360921

**Published:** 2026-11-02

**Authors:** Thanh-Lam Le, Min-Fu Hsieh

**Affiliations:** 1 Faculty of Electrical and Electronics Engineering, Ho Chi Minh City University of Technology and Engineering, Ho Chi Minh City, Vietnam; 2 Department of Electrical Engineering, National Cheng Kung University, Tainan City, Taiwan; Zhejiang University, CHINA

## Abstract

Residual tracking errors may persist in actuator-limited quadrotors after the main transient has decayed, particularly when motor lag, dead-zone behavior, thrust saturation, rotor drag, and partial motor-effectiveness loss weaken corrective action near the sliding surface. This study develops a friction-mode residual reaching law (FRMRL) for robust attitude–altitude control of a quadrotor UAV. The law retains the smoothness of a bounded sliding-mode reaching function and adds a residual-activated term that becomes effective when the sliding variable remains nonzero while the corresponding angular or vertical rate is small. It therefore strengthens the reaching action in the low-rate residual regime without increasing switching authority across the full operating range. The controller is formulated for a cascaded quadrotor architecture in which the directly actuated attitude–altitude channels are modeled as matched-input second-order subsystems subject to lumped perturbations and actuator nonlinearities. Lyapunov analysis establishes uniform ultimate boundedness under bounded perturbations and asymptotic convergence in the nominal case; it also shows a stronger local descent rate than the smooth reaching baseline in the residual regime. FRMRL is evaluated against PID, constant-rate SMC, exponential-reaching-law SMC, adaptive SMC, and a gain-matched smooth-reaching-law ablation baseline across six scenarios: nominal tracking, matched disturbances, wind-like disturbances, parameter uncertainty, actuator saturation with dead-zone, and partial motor-effectiveness loss. FRMRL achieves the lowest study-specific journal-oriented quality score in all six scenarios. Its mean score is 13.2%, 19.7%, 49.9%, and 78.4% lower than the corresponding mean scores for ERL-SMC, ASMC, CRL-SMC, and PID, respectively. Across the six scenarios, FRMRL also yields a mean total IAE of 15.5468 and a mean peak-to-peak control ripple of 0.3783. The gain-matched ablation indicates that residual activation, rather than differences in baseline gains, accounts for the improvement in residual-error suppression and control smoothness.

## 1. Introduction

Quadrotor unmanned aerial vehicles (UAVs) have become a core platform in modern aerial robotics because they combine vertical takeoff and landing, hovering capability, compact structure, and high maneuverability within a mechanically simple architecture. These features have enabled their use in infrastructure inspection, logistics and delivery, environmental monitoring, search-and-rescue missions, cooperative formation flight, target enclosing, robot-assisted landing, and industrial surface operations [1–8]. As UAVs move from controlled environments to cluttered, wind-exposed, and actuator-limited missions, the control objective has also changed. The key issue is no longer nominal stabilization around hover, but reliable tracking under aerodynamic disturbances, uncertain payloads, rotor-induced coupling, nearby-obstacle effects, energy constraints, and loss of actuation authority [9–12]. This requirement is particularly demanding for quadrotors because four rotor-generated inputs must regulate six degrees of freedom. The attitude–altitude subsystem therefore plays a central role in the cascade flight-control architecture: it produces the thrust-vector orientation and the vertical force required by the outer-loop position controller, and its robustness directly determines whether the complete UAV system can preserve tracking performance under realistic operating conditions [13,14].

The main difficulty is that the control input computed at the virtual-input level is only a nominal request. In practice, the delivered thrust and torques are modified by motor lag, thrust saturation, rotor-speed limits, dead-zone behavior, rotor drag, voltage-dependent thrust variation, and partial motor-effectiveness loss [4,15]. These actuator-side effects are often as important as aerodynamic disturbances because they enter the same channels through which the attitude–altitude controller acts. A robust control law may suppress large transient deviations, yet still leave a residual tracking error when the vehicle is already close to the desired trajectory [16]. This low-rate region is subtle but important: the angular or vertical rate can be small while the sliding variable remains nonzero. Under such a condition, the vehicle has nearly stopped moving in the controlled direction, but the remaining error is not fully removed because the actuator channel cannot deliver, or does not effectively transmit, the small corrective action required. Hence, an actuator-conscious UAV controller should not be assessed only by peak error, settling time, or nominal tracking accuracy. It should also suppress residual errors, avoid unnecessary high-frequency torque activity, and preserve smooth virtual inputs when the actuator channel contains dead-zone, saturation residuals, motor lag, or reduced effectiveness [12,17].

Several control families have been developed to address the nonlinear and uncertain nature of quadrotor flight. PID and LQR controllers remain attractive for embedded implementation because of their simple structure and low computational demand [18,19]. However, their performance is limited when the UAV operates far from the local design condition or faces significant parameter variation. Backstepping and feedback-linearization techniques improve nonlinear tracking performance by exploiting the system structure, but their effectiveness depends on the availability of a reasonably accurate model [20,21]. Model predictive control and barrier-function-based methods provide a systematic way to handle constraints, although their online optimization or constraint-management layer may increase computational burden for lightweight UAV hardware [22,23]. A recent quadrotor study has applied a PSO-tuned fractional-order adaptive model predictive control strategy to robust trajectory tracking [22]. Disturbance-observer-based control and active disturbance rejection control improve robustness by estimating lumped perturbations and compensating them in real time [24]. Adaptive, fuzzy, and neural-network-based schemes further reduce model dependence by estimating uncertain parameters, disturbance bounds, or unknown dynamics [19–26]. These methods represent an important trend in UAV control: robust tracking is increasingly formulated as an uncertainty-aware and constraint-aware problem rather than a purely nominal stabilization problem. Nevertheless, additional observers, learning modules, or adaptation laws often introduce extra tuning layers, computational cost, and implementation complexity. This motivates robust inner-loop designs that remain structurally simple, analytically transparent, and directly compatible with cascade quadrotor control.

Sliding-mode control (SMC) is well suited to this requirement because it provides strong robustness against matched uncertainties and allows the designer to shape the reaching dynamics of the sliding variable [16,24,27–29]. Classical sign-based SMC gives a strong attraction toward the sliding manifold, but the discontinuous switching term produces chattering, high-frequency torque variation, and undesirable rotor-speed activity [30]. Terminal and nonsingular terminal SMC improve transient convergence and finite-time behavior [31,32]. A recent quadrotor study combines adaptive fractional-order nonsingular terminal SMC with a wavelet neural network [10]. Super-twisting and higher-order SMC reduce the chattering associated with first-order switching while preserving robustness [33]. Adaptive SMC reduces the need for a known disturbance bound, but the adaptation mechanism introduces additional gain dynamics [34]. Observer-assisted SMC improves disturbance compensation, but the observer bandwidth, estimation error, and computational cost must be considered in practical UAV implementation [35]. Neural-network-enhanced SMC can approximate unknown dynamics or equivalent control terms, yet its performance depends on network structure, training/update laws, and real-time computational resources [36]. More recent finite-time, fixed-time, predefined-time, and prescribed-performance SMC schemes have further strengthened convergence guarantees and transient-error regulation [37,38]. These developments are valuable, but they mainly improve robustness by increasing reaching authority, estimating uncertainty, approximating the equivalent control, or redesigning the sliding surface [39]. They do not directly address a specific actuator-induced weakness of smooth reaching laws: when discontinuous switching is replaced by a bounded smooth function, the near-surface corrective action becomes weaker, and residual errors may persist under low-rate actuator nonlinearities [38,40–42].

Recent prescribed-performance and fixed-time tracking studies have also addressed explicit transient-error limits and convergence-time guarantees. Examples include an experimentally validated prescribed-performance framework for UAV flight, prescribed-performance tracking for amphibious uncrewed aerial vehicles, and fixed-time tracking for an amphibious unmanned surface vehicle [43–45]. These studies provide complementary context: they regulate transient performance or convergence time, whereas the present work focuses on low-rate, actuator-induced residual errors.

Motivated by this gap, this paper proposes a friction-mode residual reaching law (FRMRL) for robust attitude–altitude control of a quadrotor UAV. The term “friction-mode” is used in a control-oriented sense. It does not imply that the rigid-body quadrotor dynamics are governed by dry mechanical friction; rather, it denotes a low-rate residual condition produced by friction-like and actuator-induced effects such as motor dead-zone, rotor drag, thrust saturation residuals, motor lag, low-frequency bias disturbances, and partial effectiveness loss. The novelty of this work is not to make SMC more aggressive over the entire operating range, but to make the smooth reaching action selectively stronger only when a low-rate residual condition is detected. The main contributions are as follows. First, a residual-activated smooth reaching mechanism is formulated to reinforce near-surface correction when the sliding variable remains nonzero while the corresponding angular or vertical rate is small. Second, the proposed FRMRL is embedded into a cascade quadrotor architecture, where the directly actuated attitude–altitude channels are represented as matched-input second-order subsystems under lumped perturbations and actuator nonlinearities. Third, Lyapunov-based analysis is provided to establish closed-loop boundedness and to show why the proposed reaching dynamics provide a stronger local descent rate than the smooth baseline in the residual regime. Fourth, the controller is evaluated against PID, constant-rate SMC, exponential-reaching-law SMC, adaptive SMC, and a smooth-reaching-law ablation baseline. The ablation baseline, denoted as SERL-SMC, uses the same smooth base gains as the proposed FRMRL but removes the residual activation term; therefore, it isolates the effect of the proposed residual-mode correction. The benchmark scenarios include nominal tracking, matched disturbances, wind-like disturbances, parameter uncertainty, actuator saturation with dead-zone, and partial motor-effectiveness loss. The latter two cases are especially important because they directly create the low-rate residual behavior targeted by the proposed law. The results show that FRMRL improves the balance among tracking accuracy, residual-error suppression, and actuator smoothness, making it a suitable actuator-conscious reaching-law strategy for robust cascade quadrotor control.

## 2. Quadrotor modeling and problem formulation

This section develops a control-oriented model of the quadrotor UAV used for controller synthesis and comparative evaluation. The model is formulated in a cascade form, where the outer-loop position controller generates the desired roll and pitch commands, whereas the inner-loop attitude–altitude controller produces the total thrust and body torques. This formulation is particularly suitable for robust sliding-mode-based design because the directly actuated channels can be expressed as matched-input second-order subsystems.

### 2.1. Quadrotor dynamics and actuator-limited representation

Consider a quadrotor UAV treated as a rigid body with a symmetric cross configuration. Let $F_{I}=\{O_{I},x_{I},y_{I},z_{I}\}$ denote the inertial frame and $F_{B}=\{O_{B},x_{B},y_{B},z_{B}\}$ denote the body-fixed frame attached to the vehicle center of mass. The inertial position is denoted by $p=[x\;y\;z{]}^{T}$, and the attitude is described by the Euler-angle vector $\eta =[\phi \;\theta \;\psi {]}^{T}$, where ϕ, θ, and ψ are the roll, pitch, and yaw angles, respectively. The body angular velocity is $\omega ={\left[p_{b}\;q_{b}\;r_{b}\right]}^{T}$. To avoid notation conflict with the inertial position p, the body angular-rate components are denoted by $p_{b}$, $q_{b}$, and $r_{b}$. The adopted coordinate-frame convention and the representative quadrotor platform are illustrated in Fig 1.

**Fig 1:**
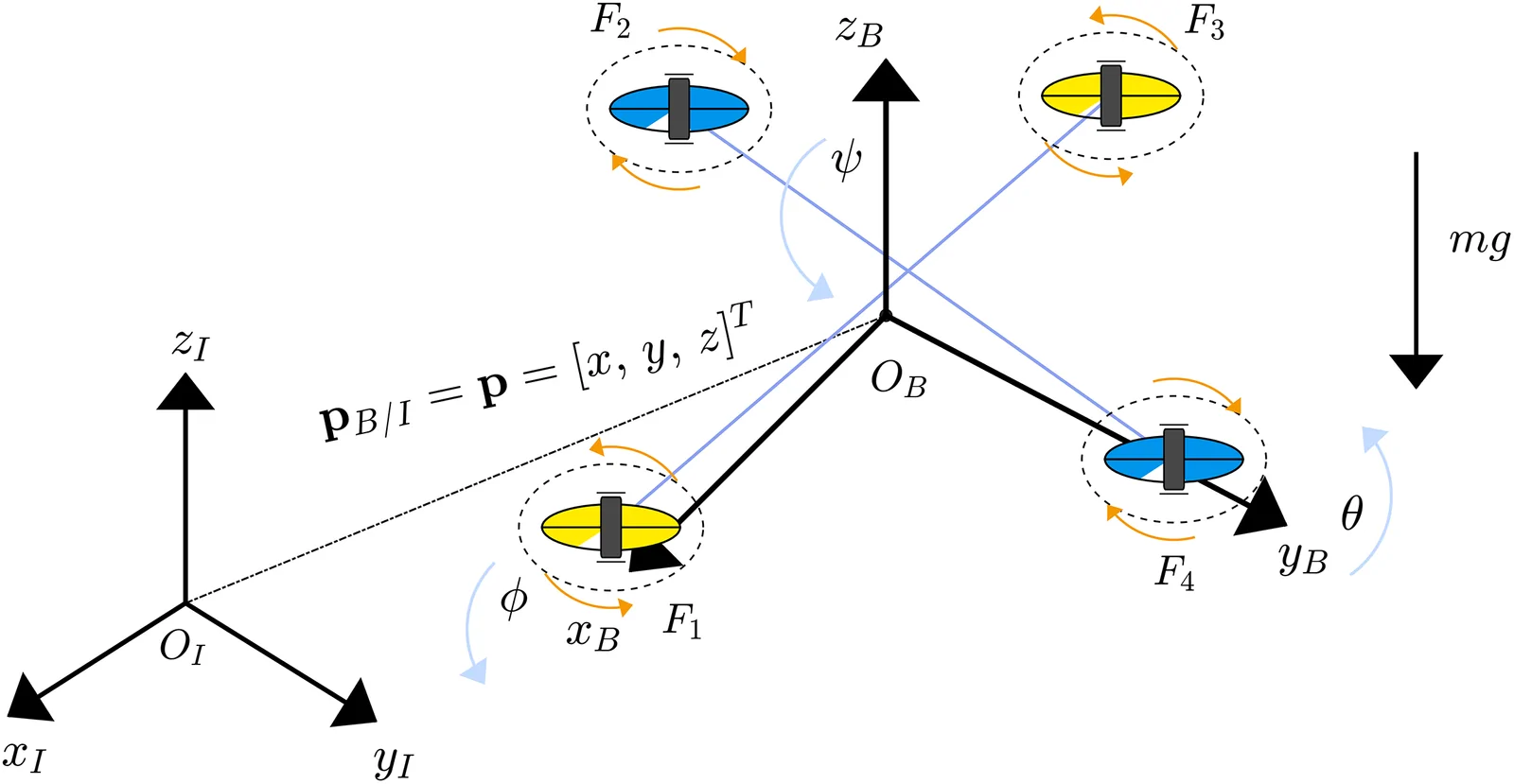
Quadrotor UAV coordinate frames and rotor thrusts.

The translational and rotational dynamics can be written in compact Newton–Euler form as


$$\begin{array}{c} m\ddot{p}=-mge_{\text{3}}+R(\eta )e_{\text{3}}U_{\text{1}}+d_{p}(t),\;\;J\dot{\omega }=-\omega \times J\omega +\tau +\tau_{g}+d_{\eta }(t) \end{array}$$ (1)

where m is the quadrotor mass, g is the gravitational acceleration, $e_{\text{3}}=[\text{0}\;\text{0}\;\text{1}{]}^{T}$, R(η)∈SO(3) is the rotation matrix from $F_{B}$ to $F_{I}$, $U_{\text{1}}$ is the total thrust, $J=\text{diag}\left(I_{x},I_{y},I_{z}\right)$ is the inertia matrix, $\tau ={\left[U_{\text{2}}\;U_{\text{3}}\;U_{\text{4}}\right]}^{T}$ is the vector of roll, pitch, and yaw control torques, $\tau_{g}$ denotes the gyroscopic torque induced by rotor rotation, and $d_{p}(t)$ and $d_{\eta }(t)$ collect aerodynamic disturbances, wind-gust effects, unmodeled dynamics, and actuator-induced uncertainties.

For controller design, the attitude–altitude subsystem is expressed in scalar form as


$$\begin{array}{c} \begin{array}{cc} \ddot{\phi } & =\frac{I_{y}-I_{z}}{I_{x}}\dot{\theta }\dot{\psi }-\frac{J_{r}}{I_{x}}\dot{\theta }\Omega_{r}+\frac{1}{I_{x}}U_{2}+\Delta_{\phi }(t),\\ \ddot{\theta } & =\frac{I_{z}-I_{x}}{I_{y}}\dot{\phi }\dot{\psi }+\frac{J_{r}}{I_{y}}\dot{\phi }\Omega_{r}+\frac{1}{I_{y}}U_{3}+\Delta_{\theta }(t),\\ \ddot{\psi } & =\frac{I_{x}-I_{y}}{I_{z}}\dot{\phi }\dot{\theta }+\frac{1}{I_{z}}U_{4}+\Delta_{\psi }(t),\\ \ddot{z} & =\frac{U_{1}}{m}\cos\phi \cos\theta -g+\Delta_{z}(t). \end{array} \end{array}$$ (2)

Here, $J_{r}$ is the rotor moment of inertia, $\Omega_{r}$ is the residual rotor-speed imbalance term, and $\Delta_{\phi }(t)$, $\Delta_{\theta }(t)$, $\Delta_{\psi }(t)$, and $\Delta_{z}(t)$ denote matched lumped perturbations in the roll, pitch, yaw, and altitude channels. These terms include external disturbances, parametric deviations, neglected aerodynamic effects, actuator nonlinearities, and imperfect thrust generation.

The horizontal translational dynamics are not directly actuated. They are given by


$$\begin{array}{c} \begin{array}{cc} \ddot{x} & =\frac{U_{\text{1}}}{m}(\text{cos}\phi \text{sin}\theta \text{cos}\psi +\text{sin}\phi \text{sin}\psi )+\Delta_{x}(t),\\ \ddot{y} & =\frac{U_{\text{1}}}{m}(\text{cos}\phi \text{sin}\theta \text{sin}\psi -\text{sin}\phi \text{cos}\psi )+\Delta_{y}(t), \end{array} \end{array}$$ (3)

where $\Delta_{x}(t)$ and $\Delta_{y}(t)$ are the lumped horizontal disturbances. Equations (2) and (3) reveal the underactuated nature of the quadrotor: x and y are influenced through the attitude angles and the total thrust, whereas z, ϕ, θ, and ψ are directly associated with $U_{\text{1}}$, $U_{\text{2}}$, $U_{\text{3}}$, and $U_{\text{4}}$.

The virtual control inputs are generated from the four rotor speeds according to the standard motor-mixing relation


$$\begin{array}{c} {}[\begin{array}{c} U_{\text{1}}\\ U_{\text{2}}\\ U_{\text{3}}\\ U_{\text{4}}\\ \Omega_{r} \end{array}]=[\begin{array}{c} b\left(\Omega_{\text{1}}^{\text{2}}+\Omega_{\text{2}}^{\text{2}}+\Omega_{\text{3}}^{\text{2}}+\Omega_{\text{4}}^{\text{2}}\right)\\ bl\left(\Omega_{\text{4}}^{\text{2}}-\Omega_{\text{2}}^{\text{2}}\right)\\ bl\left(\Omega_{\text{1}}^{\text{2}}-\Omega_{\text{3}}^{\text{2}}\right)\\ d\left(-\Omega_{\text{1}}^{\text{2}}+\Omega_{\text{2}}^{\text{2}}-\Omega_{\text{3}}^{\text{2}}+\Omega_{\text{4}}^{\text{2}}\right)\\ -\Omega_{\text{1}}+\Omega_{\text{2}}-\Omega_{\text{3}}+\Omega_{\text{4}} \end{array}], \end{array}$$ (4)

where b is the thrust coefficient, d is the drag coefficient, l is the arm length, and $\Omega_{i}$ is the angular speed of the i-th rotor. The signs in (4) follow the adopted rotor numbering and rotation convention; a different numbering convention only changes the corresponding mixer matrix, not the control structure.

In a practical quadrotor, the ideal command $U={\left[U_{\text{1}}\;U_{\text{2}}\;U_{\text{3}}\;U_{\text{4}}\right]}^{T}$ is not applied directly to the rigid-body dynamics. Motor lag, thrust saturation, rotor-speed limits, dead-zone effects, and partial loss of actuator effectiveness modify the actual input delivered to the vehicle. To retain a tractable but non-ideal simulation model, the rotor dynamics are represented by


$$\begin{array}{c} \tau_{m}{\dot{\Omega }}_{i}=-\Omega_{i}+\text{sat}\left(\Omega_{i,c}\right),\;\;\Omega_{i,e}=\sqrt{\eta_{i}}D\left(\Omega_{i}\right) \end{array}$$ (5)

where $\tau_{m}$ is the motor time constant, $\Omega_{i,c}$ is the commanded rotor speed, $\Omega_{i,e}$ is the effective rotor speed, $\eta_{i}\in (\text{0},\text{1}]$ is the actuator effectiveness factor, and D(·) denotes a dead-zone map. In this work, the dead-zone is modeled as $D\left(\Omega_{i}\right)=\text{0}$ for $\left|\Omega_{i}\right|<\Omega_{dz}$, and $D\left(\Omega_{i}\right)=\Omega_{i}-\Omega_{dz}\text{sgn}\left(\Omega_{i}\right)$ otherwise, where $\Omega_{dz}$ is the rotor-speed dead-zone threshold.

Because the mixer in Eq. (4) uses squared rotor speeds, actuator effectiveness scales each rotor's thrust and drag-torque contribution directly. Equation (5) therefore uses $\sqrt{\eta_{\text{i}}}$ as an equivalent speed factor, so that after dead-zone mapping $\Omega_{i}{,}_{e}^{\text{2}}\;=\;\eta_{i}\;[\text{D}(\Omega_{i}){]}^{\text{2}}$ matches the effectiveness-weighted squared speed used by the simulation.

The corresponding effective input $U_{e}={\left[U_{\text{1},e}\;U_{\text{2},e}\;U_{\text{3},e}\;U_{\text{4},e}\right]}^{T}$ is obtained by replacing $\Omega_{i}$ in (4) with $\Omega_{i,e}$. The difference between $U_{e}$ and the nominally commanded virtual input is treated as a matched input perturbation. Consequently, the directly actuated channels can be written in the following unified form:


$$\begin{array}{c} {\ddot{y}}_{i}=f_{i}(X)+g_{i}(X)u_{i}+\Delta_{i}(t),\;i\in 𝒥:=\{\phi ,\theta ,\psi ,z\} \end{array}$$ (6)

where $X={\left[\phi ,\dot{\phi },\theta ,\dot{\theta },\psi ,\dot{\psi },z,\dot{z},x,\dot{x},y,\dot{y}\right]}^{T}$ is the state vector, $y_{i}$ is the controlled output of the i-th channel, and $u_{i}$ denotes the corresponding virtual control input. Specifically, $u_{\phi }=U_{\text{2}}$, $u_{\theta }=U_{\text{3}}$, $u_{\psi }=U_{\text{4}}$, and $u_{z}=U_{\text{1}}$. The functions $f_{i}(X)$ contain the known nominal nonlinear coupling terms, $g_{i}(X)$ are the input gains, and $\Delta_{i}(t)$ are the matched lumped perturbations.

For the four controlled channels, the nominal drift and input-gain functions are


$$\begin{array}{cc} f_{\phi } & =\frac{I_{y}-I_{z}}{I_{x}}\dot{\theta }\dot{\psi }-\frac{J_{r}}{I_{x}}\dot{\theta }\Omega_{r},\;g_{\phi }=\frac{1}{I_{x}}\\ f_{\theta } & =\frac{I_{z}-I_{x}}{I_{y}}\dot{\phi }\dot{\psi }+\frac{J_{r}}{I_{y}}\dot{\phi }\Omega_{r},\;g_{\theta }=\frac{1}{I_{y}}\\ f_{\psi } & =\frac{I_{x}-I_{y}}{I_{z}}\dot{\phi }\dot{\theta },\;\;\;g_{\psi }=\frac{1}{I_{z}}\\ f_{z} & =-g,\;\;g_{z}=\frac{\cos\phi \cos\theta }{m} \end{array}$$ (7)

This affine representation is the basis for the proposed reaching-law-based sliding-mode controller developed in the next section.

### 2.2. Cascade architecture and sliding-variable formulation

The quadrotor has six degrees of freedom but only four independent control inputs. Therefore, full position and attitude regulation must be handled through a cascade architecture. The outer position loop regulates x and y indirectly by generating the desired roll and pitch angles, whereas the inner loop tracks $\phi_{d}$, $\theta_{d}$, $\psi_{d}$, and $z_{d}$.

Let $a_{x}^{d}$, $a_{y}^{d}$, and $a_{z}^{d}$ be the desired translational accelerations generated by the outer-loop position controller. For a given desired yaw angle $\psi_{d}$, the desired total thrust magnitude and attitude commands can be obtained from


$$\begin{array}{c} T_{d}=m\sqrt{{\left(a_{x}^{d}\right)}^{\text{2}}+{\left(a_{y}^{d}\right)}^{\text{2}}+{\left(a_{z}^{d}+g\right)}^{\text{2}}},\;\phi_{d}={\text{sin}}^{-\text{1}}\left(\frac{m}{T_{d}}\left(a_{x}^{d}\text{sin}\psi_{d}-a_{y}^{d}\text{cos}\psi_{d}\right)\right), \end{array}$$ (8)

$$\begin{array}{c} \theta_{d}={\text{tan}}^{-\text{1}}\left(\frac{a_{x}^{d}\text{cos}\psi_{d}+a_{y}^{d}\text{sin}\psi_{d}}{a_{z}^{d}+g}\right). \end{array}$$ (9)

Equations (8) and (9) formalize the conversion block in the cascade architecture. In near-hover operation, they reduce to the familiar approximation $\theta_{d}\approx \left(a_{x}^{d}\text{cos}\psi_{d}+a_{y}^{d}\text{sin}\psi_{d}\right)/g$ and $\phi_{d}\approx \left(a_{x}^{d}\text{sin}\psi_{d}-a_{y}^{d}\text{cos}\psi_{d}\right)/g$. The proposed controller is designed for the inner attitude–altitude loop; the outer-loop position controller is retained to provide feasible references and to preserve the standard cascade structure used in practical UAV autopilots.

The overall cascade control structure of the actuator-limited quadrotor UAV is summarized in Fig 2, where the outer position loop generates the attitude–altitude references and the proposed FRMRL is applied only to the inner attitude–altitude loop.

**Fig 2:**
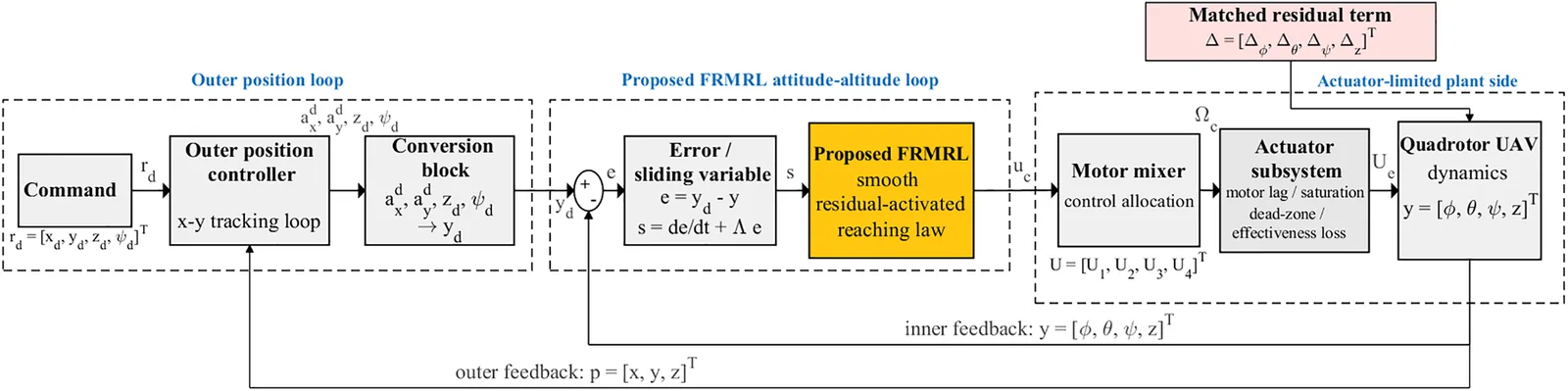
Cascade control structure of the actuator-limited quadrotor UAV with the proposed FRMRL attitude–altitude loop.

**Remark 1.** The proposed controller should not be interpreted as a direct controller for all six outputs (x,y,z,ϕ,θ,ψ). It directly regulates z, ϕ, θ, and ψ. The horizontal positions x and y are regulated indirectly through the commanded attitude angles $\phi_{d}$ and $\theta_{d}$. This distinction is essential because the quadrotor is underactuated, and any claim of full position tracking must be understood in the cascade sense.

The present analysis and simulations concern the inner attitude–altitude loop. Errors or delays in the outer position loop are not modeled separately; they would enter through the generated desired roll, pitch, and thrust references and could degrade inner-loop tracking if those references are not sufficiently smooth and bounded or exceed the actuator-feasible range. Because no outer-loop reference-error or delay sweep was performed, the present results do not quantify this sensitivity.

For the inner-loop subsystem, define the tracking errors as


$$\begin{array}{c} e_{i}=y_{i}-y_{i,d},\;\;{\dot{e}}_{i}={\dot{y}}_{i}-{\dot{y}}_{i,d},\;\;i\in 𝒥, \end{array}$$ (10)

where $y_{\phi ,d}=\phi_{d}$, $y_{\theta ,d}=\theta_{d}$, $y_{\psi ,d}=\psi_{d}$, and $y_{z,d}=z_{d}$. The first-order sliding variable is selected as


$$\begin{array}{c} s_{i}={\dot{e}}_{i}+\lambda_{i}e_{i},\;\;\lambda_{i}>\text{0}. \end{array}$$ (11)

When $s_{i}=\text{0}$, the tracking error satisfies ${\dot{e}}_{i}+\lambda_{i}e_{i}=\text{0}$, which implies exponential convergence of $e_{i}$ to zero in the ideal sliding motion. Therefore, the controller design objective is reduced to enforcing a stable reaching dynamics for $s_{i}$ under matched perturbations and actuator nonlinearities.

### 2.3. Control objective and standing assumptions

**Assumption 1.** The desired inner-loop trajectories $y_{i,d}$, ${\dot{y}}_{i,d}$, and ${\ddot{y}}_{i,d}$ are continuous and bounded for all i∈I.

**Assumption 2.** The roll and pitch angles remain inside a compact nonsingular operating set, i.e., there exists a constant $\varepsilon_{\eta }>\text{0}$ such that $|\phi |<\pi /\text{2}-\varepsilon_{\eta }$ and $|\theta |<\pi /\text{2}-\varepsilon_{\eta }$. Consequently, cosϕcosθ is lower bounded by a positive constant.

Assumptions 1 and 2 define a local operating envelope for the inner attitude–altitude loop. They require the commanded trajectories and the reference derivatives used by the model to remain bounded, and the roll and pitch angles to stay a positive margin away from the Euler-angle singularity at ±90°. The analysis therefore covers feasible, locally regulated attitude–altitude trajectories within this nonsingular region; it does not establish tracking for near-vertical attitudes or arbitrary aggressive maneuvers. Because this study does not identify a platform-specific maximum tilt angle, no numerical flight-envelope limit is inferred.

**Assumption 3.** The lumped perturbations $\Delta_{i}(t)$ are bounded but not necessarily known. That is, there exist unknown positive constants ${\bar{\Delta }}_{i}$ such that $\left|\Delta_{i}(t)\right|\leq {\bar{\Delta }}_{i}$ for all t≥0 and all i∈I.

**Assumption 4.** The actuator effectiveness factors satisfy $\text{0}<\eta_{\text{min}}\leq \eta_{i}\leq \text{1}$. The actuator dead-zone and saturation residuals are bounded and enter the attitude–altitude dynamics through the same channels as the virtual control inputs.

**Remark 2.** Assumption 3 is standard in robust sliding-mode control and does not require exact knowledge of the disturbance upper bound in the simulation model. Assumption 4 is introduced to support a more realistic validation of the proposed reaching law. In particular, it allows low-rate residual errors caused by actuator dead-zone, motor lag, rotor drag, or partial effectiveness loss to be represented as matched perturbations in the inner-loop dynamics.

The proposed reaching law in the next section employs smooth bounded functions instead of discontinuous switching. The following elementary property will be used in the stability analysis.

**Lemma 1.** For any δ>0 and any scalar s∈R, the function tanh(s/δ) satisfies


$$\begin{array}{c} s\text{tanh}\left(\frac{s}{\delta }\right)\geq \text{0},\;\;\left|\text{tanh}\left(\frac{s}{\delta }\right)\right|\leq \text{1}. \end{array}$$ (12)

**Proof.** Since tanh(·) is an odd and monotonically increasing function, tanh(s/δ) has the same sign as s. Hence, their product is nonnegative. The boundedness follows directly from the range of the hyperbolic tangent function.

The objective of this work is to design a smooth robust inner-loop controller for the attitude–altitude subsystem of the quadrotor UAV. The controller must generate $U_{\text{1}}$, $U_{\text{2}}$, $U_{\text{3}}$, and $U_{\text{4}}$ such that the controlled outputs z, ϕ, θ, and ψ track their references $z_{d}$, $\phi_{d}$, $\theta_{d}$, and $\psi_{d}$ despite nonlinear coupling, parametric uncertainty, external disturbances, actuator saturation, dead-zone effects, and partial loss of actuator effectiveness.

Formally, for the system (6), the control problem is to construct $u_{i}$ for all i∈I such that all closed-loop signals remain bounded and


$$\begin{array}{c} \underset{t\to \infty }{\text{limsup}}\left|e_{i}(t)\right|\leq \rho_{i},\;\;\underset{t\to \infty }{\text{limsup}}\left|s_{i}(t)\right|\leq \sigma_{i}, \end{array}$$ (13)

where $\rho_{i}$ and $\sigma_{i}$ are small tunable residual bounds. In the absence of lumped perturbations and actuator nonlinearities, the residual bounds should reduce to zero. In addition, the resulting control inputs should remain continuous or practically smooth to avoid high-frequency chattering and excessive actuator activity.

The key design challenge is that a conventional sign-based sliding-mode controller can improve robustness but usually induces chattering, whereas a purely smooth reaching law may lose residual-error suppression capability near low-rate operating conditions. Therefore, the next section introduces a low-rate residual-activated smooth reaching law, in which the reaching effort is selectively strengthened when the sliding variable remains nonzero while the corresponding angular or vertical rate becomes small. This mechanism is intended to improve steady residual-error suppression under actuator dead-zone and friction-like unmodeled effects without sacrificing the smoothness of the control action.

## 3. Proposed friction-mode residual reaching law and controller design

This section develops the proposed control law for the attitude–altitude subsystem of the quadrotor UAV. The design is carried out in two steps. First, the limitations of conventional sign-based and smooth reaching laws are analyzed to clarify the need for a new reaching mechanism. Second, the proposed friction-mode residual reaching law is embedded into the quadrotor inner-loop controller, and the closed-loop stability is established using Lyapunov analysis.

### 3.1. From sign-based sliding mode to residual-activated reaching

For the controlled channel


$$\begin{array}{c} {\ddot{y}}_{i}=f_{i}(X)+g_{i}(X)u_{i}+\Delta_{i}(t),\;\;i\in 𝒥:=\left\{\phi ,\theta ,\psi ,z\right\}, \end{array}$$ (14)

with the sliding variable $s_{i}={\dot{e}}_{i}+\lambda_{i}e_{i}$, the central task of sliding-mode control is to impose a stable reaching dynamics for $s_{i}$. The most direct choice is the sign-based reaching law


$$\begin{array}{c} {\dot{s}}_{i}=-\alpha_{i}\text{sgn}\left(s_{i}\right),\;\;\alpha_{i}>\text{0}. \end{array}$$ (15)

The appeal of (15) is its strong robustness. In the nominal case, $s_{i}$ reaches the sliding manifold in finite time. Indeed, with $V_{i}=s_{i}^{\text{2}}/\text{2}$, one obtains ${\dot{V}}_{i}=-\alpha_{i}\left|s_{i}\right|$, which implies finite-time convergence. However, the same discontinuity that guarantees strong reaching also produces high-frequency switching. In a quadrotor, this switching is propagated through the motor mixer to rotor-speed commands, where it may excite actuator dynamics, amplify measurement noise, and increase the total variation of the thrust and torque commands.

A widely used improvement is the exponential reaching law


$$\begin{array}{c} {\dot{s}}_{i}=-k_{s,i}s_{i}-\alpha_{i}\text{sgn}\left(s_{i}\right),\;\;k_{s,i}>\text{0}. \end{array}$$ (16)

The linear term accelerates the decay when $\left|s_{i}\right|$ is large, whereas the sign term preserves robustness against matched perturbations. Nevertheless, the discontinuous component remains. Replacing $\text{sgn}\left(s_{i}\right)$ with a smooth bounded function gives


$$\begin{array}{c} {\dot{s}}_{i}=-k_{s,i}s_{i}-k_{c,i}\text{tanh}\left(\frac{s_{i}}{\delta_{i}}\right),\;\;k_{c,i}>\text{0},\;\delta_{i}>\text{0}, \end{array}$$ (17)

where $k_{c,i}$ is the smooth robust gain and $\delta_{i}$ determines the thickness of the smooth boundary layer. The law in (17) significantly reduces chattering, but it also changes the behavior near the origin. Since $\text{tanh}\left(s_{i}/\delta_{i}\right)\approx s_{i}/\delta_{i}$ when $\left|s_{i}\right|$ is small, the discontinuous robust action is replaced by a linear-like correction around the sliding surface. This is beneficial for smoothness, but it may leave a persistent residual error when the system is affected by actuator dead-zone, rotor drag, motor lag, partial effectiveness loss, or slowly varying bias disturbances.

This weakness is particularly important in quadrotor attitude–altitude control. During aggressive transients, the sliding variable is large and all reaching laws can generate sufficient corrective effort. The more difficult regime occurs when the corresponding angular or vertical rate is already small, but the sliding variable remains nonzero. In that case, the vehicle has nearly stopped moving in the controlled direction, yet the tracking error has not been fully removed. A purely smooth reaching law may not supply enough additional effort to break this residual condition unless the nominal gain is increased everywhere, which in turn increases control activity during the entire response.

To address this issue, this work introduces a friction-mode residual reaching law. The term “friction-mode” is used in a control-oriented sense. It does not imply that the rigid-body quadrotor is dominated by dry mechanical friction. Rather, it refers to a low-rate residual regime produced by friction-like and actuator-induced effects, including rotor drag, motor dead-zone, thrust saturation residuals, and low-frequency bias disturbances.

### 3.2. Proposed friction-mode residual reaching law

Define the smooth switching function


$$\begin{array}{c} \Phi_{i}\left(s_{i}\right)=\text{tanh}\left(\frac{s_{i}}{\delta_{i}}\right), \end{array}$$ (18)

where $\delta_{i}>\text{0}$. To detect the low-rate residual regime, introduce the activation factor


$$\begin{array}{c} \chi_{i}\left(s_{i},\nu_{i}\right)=\text{exp}\left(-\frac{\left|\nu_{i}\right|}{v_{i}}\right)\frac{\left|s_{i}\right|}{\left|s_{i}\right|+\sigma_{i}}, \end{array}$$ (19)

where $\nu_{i}$ is the physical rate of the i-th channel, $v_{i}>\text{0}$ is the rate-sensitivity coefficient, and $\sigma_{i}>\text{0}$ is the residual threshold. Specifically, $\nu_{\phi }=\dot{\phi }$, $\nu_{\theta }=\dot{\theta }$, $\nu_{\psi }=\dot{\psi }$, and $\nu_{z}=\dot{z}$.

The proposed friction-mode residual reaching law is


$$\begin{array}{c} {\dot{s}}_{i}=-k_{s,i}s_{i}-\left(k_{c,i}+k_{r,i}\chi_{i}\right)\Phi_{i}\left(s_{i}\right), \end{array}$$ (20)

where $k_{s,i}>\text{0}$, $k_{c,i}>\text{0}$, and $k_{r,i}>\text{0}$. The gain $k_{r,i}$ is not a conventional switching gain. It is activated through $\chi_{i}$, and therefore it contributes mainly when $\left|\nu_{i}\right|$ is small while $\left|s_{i}\right|$ remains non-negligible.

**Practical parameter-selection guideline.** Characterize each rotor dead-zone and the largest motor time constant expected in operation. With the mixer and actuator model, identify the low-rate residual errors these effects can leave; use the sliding-surface definition to express the corresponding tolerated error in sliding-variable units and set the residual threshold on that scale. In Eq. (19), the state factor is one-half when the sliding-variable magnitude equals the threshold. Next, sweep physical rates with the same dead-zone and worst-case lag, and choose the rate-sensitivity coefficient as the rate at which the exponential gate falls to exp(−1). Set the boundary-layer width above measured sliding-variable noise but within the tolerated residual band. Increase the residual gain only until low-rate residual error is removed without sustained input saturation, and select the baseline gains to meet the transient and disturbance margins in Theorem 1. Verify that activation is attenuated during fast transients but effective when a low-rate residual persists. These channel-specific criteria provide an initial setting, not universal values.

**Definition 1.** The i-th channel is said to be in a low-rate residual regime if $\left|\nu_{i}\right|\leq \nu_{i}^{⋆}$ and $\left|s_{i}\right|\geq s_{i}^{⋆}$, where $\nu_{i}^{⋆}>\text{0}$ and $s_{i}^{⋆}>\text{0}$ are prescribed small constants. This regime describes a condition in which the output motion has nearly stalled but the sliding variable has not yet been sufficiently attenuated.

**Lemma 2.** For all $s_{i},\nu_{i}\in \text{R}$, the activation factor in (19) satisfies $\text{0}\leq \chi_{i}\left(s_{i},\nu_{i}\right)<\text{1}$. Moreover, in the low-rate residual regime,


$$\begin{array}{c} \chi_{i}\left(s_{i},\nu_{i}\right)\geq {\underset{―}{\chi }}_{i}:=\text{exp}\left(-\frac{\nu_{i}^{⋆}}{v_{i}}\right)\frac{s_{i}^{⋆}}{s_{i}^{⋆}+\sigma_{i}}. \end{array}$$ (21)

**Proof.** Since $\text{exp}\left(-\left|\nu_{i}\right|/v_{i}\right)\in (\text{0},\text{1}]$ and $\left|s_{i}\right|/\left(\left|s_{i}\right|+\sigma_{i}\right)\in [\text{0},\text{1})$, the first statement follows directly. If $\left|\nu_{i}\right|\leq \nu_{i}^{⋆}$ and $\left|s_{i}\right|\geq s_{i}^{⋆}$, then $\text{exp}\left(-\left|\nu_{i}\right|/v_{i}\right)\geq \text{exp}\left(-\nu_{i}^{⋆}/v_{i}\right)$ and $\left|s_{i}\right|/\left(\left|s_{i}\right|+\sigma_{i}\right)\geq s_{i}^{⋆}/\left(s_{i}^{⋆}+\sigma_{i}\right)$. This proves (21).

**Proposition 1.** In the disturbance-free reaching dynamics, the proposed law in (20) provides a Lyapunov decay rate that is never weaker than that of the smooth reaching law in (17). The decay is strictly stronger whenever $s_{i}\neq \text{0}$ and $\chi_{i}>\text{0}$.

**Proof.** Let $V_{i}=s_{i}^{\text{2}}/\text{2}$. Under the smooth reaching law (17),


$$\begin{array}{c} {\dot{V}}_{i,\text{sm}}=-k_{s,i}s_{i}^{\text{2}}-k_{c,i}s_{i}\Phi_{i}\left(s_{i}\right). \end{array}$$ (22)

Under the proposed law (20),


$$\begin{array}{c} {\dot{V}}_{i,\text{pr}}=-k_{s,i}s_{i}^{\text{2}}-\left(k_{c,i}+k_{r,i}\chi_{i}\right)s_{i}\Phi_{i}\left(s_{i}\right). \end{array}$$ (23)

Since $s_{i}\Phi_{i}\left(s_{i}\right)\geq \text{0}$ and $k_{r,i}\chi_{i}\geq \text{0}$,


$$\begin{array}{c} {\dot{V}}_{i,\text{pr}}-{\dot{V}}_{i,\text{sm}}=-k_{r,i}\chi_{i}s_{i}\Phi_{i}\left(s_{i}\right)\leq \text{0}. \end{array}$$ (24)

The inequality is strict when $s_{i}\neq \text{0}$ and $\chi_{i}>\text{0}$.

**Remark 3.** Proposition 1 is important for the interpretation of the proposed law. The proposed reaching law is not simply a smoothed version of sign-based SMC. It contains the smooth law in (17) as a special case when $k_{r,i}=\text{0}$, and adds a bounded state-dependent gain that improves the descent rate specifically in residual regimes.

### 3.3. Mechanism and numerical assessment of the proposed reaching law

The proposed reaching law can be interpreted by separating the reaching behavior into three regimes. When $\left|s_{i}\right|$ is large, the dominant action comes from $-k_{s,i}s_{i}$ and the saturated value of $\Phi_{i}\left(s_{i}\right)$, which gives a fast transient response. When $\left|s_{i}\right|$ is small, the rational factor in $\chi_{i}$ attenuates the residual gain, preventing unnecessary amplification around the sliding surface. When $\left|\nu_{i}\right|$ is small but $\left|s_{i}\right|$ is still non-negligible, the residual activation mechanism detects a low-rate residual condition and increases the smooth reaching gain through $k_{r,i}\chi_{i}$.

This mechanism directly addresses a weakness of conventional smooth SMC. Increasing $k_{c,i}$ globally can improve residual rejection, but it also increases control effort during the full transient response. In contrast, the proposed reaching law introduces additional corrective effort only in the residual regime. As a result, it preserves smoothness during normal operation while improving robustness when actuator nonlinearities or low-frequency disturbances prevent complete error removal. To support this interpretation, Fig 3 summarizes the reaching and sliding mechanisms associated with classical SMC and the proposed FRMRL. The sign-type reaching law enforces an aggressive attraction toward the sliding manifold, but its discontinuity produces switching activity near s=0. In contrast, the proposed FRMRL retains a smooth convergence profile and increases the reaching effort only in the low-rate residual region, thereby improving residual-error suppression without reintroducing discontinuous switching.

**Fig 3:**
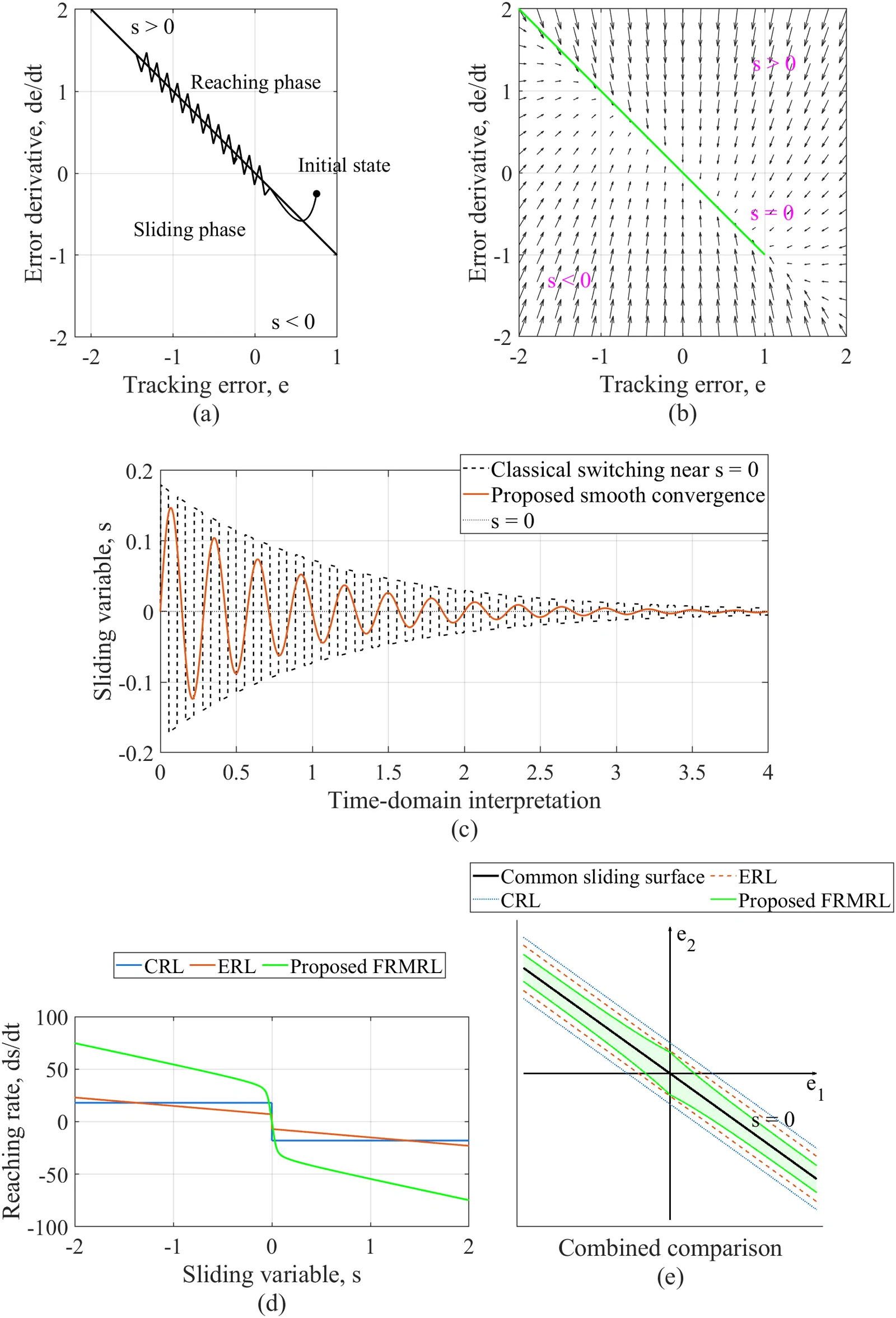
Control-oriented interpretation of classical SMC and the proposed FRMRL: (a) classical reaching and sliding motion in the $(e,\dot{e})$ phase plane; (b) vector-field attraction toward the sliding manifold s=0; (c) switching behavior of a sign-type reaching law and smooth convergence of the proposed law near s=0; (d) reaching-rate comparison among CRL, ERL, and the proposed FRMRL; and (e) phase-plane interpretation of the common sliding surface and the corresponding quasi-sliding layers.

Before evaluating the complete quadrotor control system, the intrinsic behavior of the proposed FRMRL is examined on a scalar second-order SMC-controlled channel. This intermediate test is introduced to isolate the reaching-law effect from the outer-loop trajectory generation, motor mixing, and multi-axis coupling of the UAV. The controlled channel is represented by $\ddot{y}=-a_{d}\dot{y}+bu+d_{r}(t);$ where y is the controlled output, u is the bounded control input, and $d_{r}(t)$ denotes a residual low-rate perturbation. The sliding variable is selected as $s=\dot{e}+\lambda e$; with $e=y-y_{d}$. Three reaching laws are compared under the same plant, reference, initial condition, disturbance profile, and input limit: the constant-rate law (CRL), the exponential reaching law (ERL), and the proposed FRMRL. The numerical setting uses $a_{d}=\text{25}$, b=133, $u_{\text{max}}=\text{50}$, λ=15, T=5s, and a sampling step of ${\text{10}}^{-\text{4}}\;\text{s}$. The initial condition is y(0)=−2, $\dot{y}(\text{0})=-\text{2}$, and the residual perturbation is enabled with amplitude parameter $A_{r}=\text{2}.\text{4}$, rate parameter $v_{r}=\text{0}.\text{2}$, and smoothing width $\delta_{r}=\text{0}.\text{05}$. The adopted reaching-law parameters are $\alpha_{\text{CRL}}=\text{18}$, $k_{s,\text{ERL}}=\text{8}$, $\alpha_{\text{ERL}}=\text{7}$, and, for the proposed FRMRL, $k_{s}=\text{20}$, $k_{c}=\text{16}$, $k_{r}=\text{20}$, δ=0.035, $v_{\text{rate}}=\text{0}.\text{6}$, and σ=0.03.

Fig 4 compares the tracking error, zoomed steady-state error, control input, normalized indices, phase-plane motion, and residual activation behavior of the three reaching laws. The CRL generates a strong reaching action but produces a highly oscillatory control signal because of the discontinuous switching term. The ERL reduces the switching severity, but its near-surface correction becomes weak when the trajectory approaches the sliding manifold, leading to a slower residual decay. In contrast, the proposed FRMRL keeps the control signal smooth while activating an additional reaching effort only when the system enters the low-rate residual region. This explains the faster attenuation of e and s, together with the visibly smaller control-input variation.

**Fig 4:**
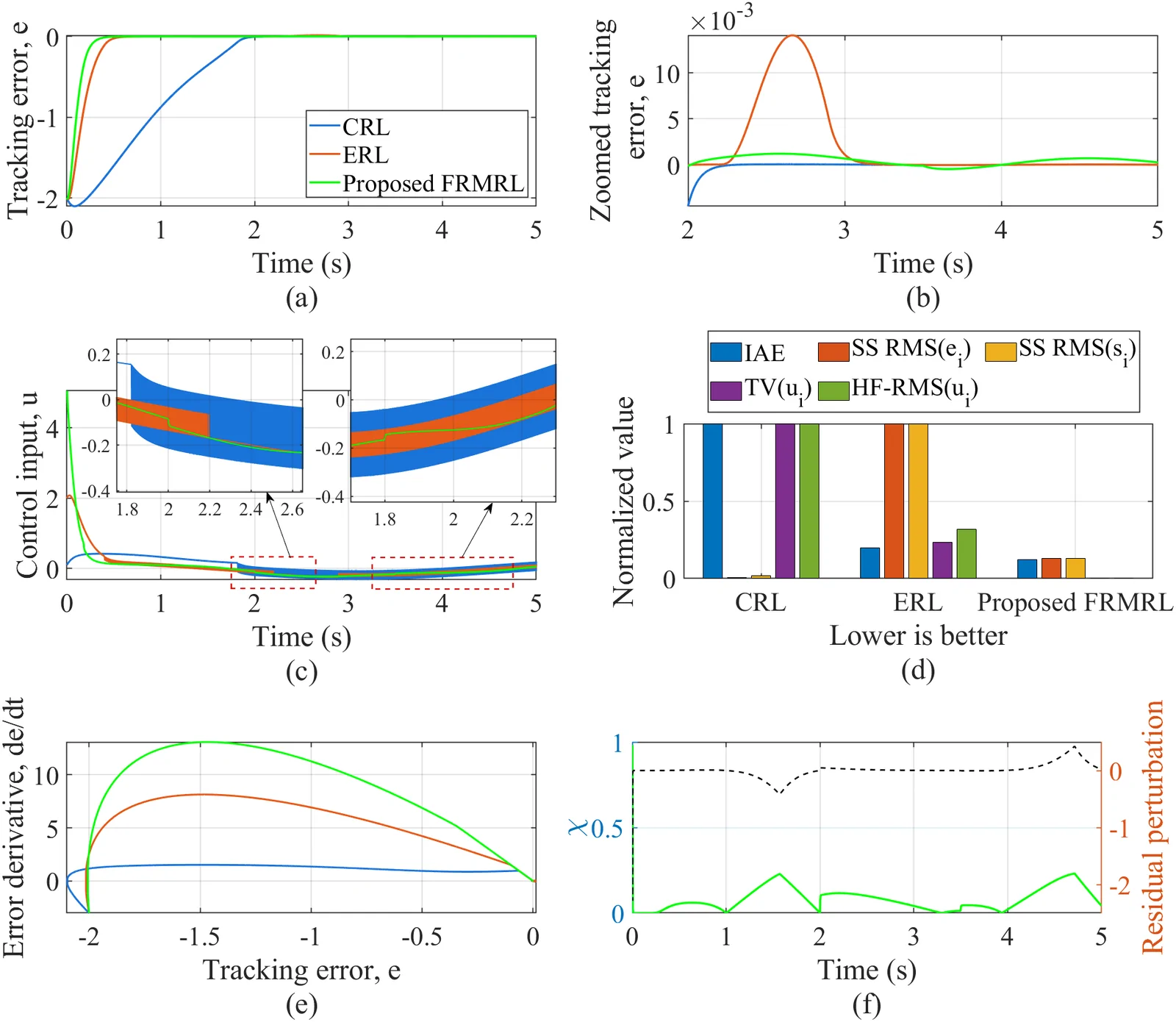
Reaching-law response comparison for an SMC-controlled second-order channel: (a) tracking-error response, (b) zoomed steady-state tracking error, (c) control input with enlarged residual-region insets, (d) normalized performance indices, (e) phase-plane trajectory in the $\left(e,\dot{e}\right)$ plane, and (f) residual activation factor and residual perturbation profile.

The quantitative comparison confirms the behavior observed in Fig 4. The proposed FRMRL gives the lowest RMSE, IAE, ITAE, control RMS, control total variation, high-frequency control RMS, reaching time, and settling time among the compared methods. Relative to CRL, the RMSE, IAE, ITAE, control total variation, high-frequency control RMS, reaching time, and settling time are reduced by 67.34%, 87.92%, 97.78%, 99.99%, 99.86%, 90.09%, and 74.79%, respectively. Relative to ERL, the corresponding reductions are 22.46%, 38.51%, 60.22%, 99.98%, 99.57%, 93.74%, and 82.46%, respectively. Although the CRL can yield a very small steady-state sliding-variable RMS in the nominal scalar test, this is achieved at the cost of excessive high-frequency control activity. The proposed FRMRL therefore provides a more balanced reaching behavior: fast convergence, low residual error, and substantially smoother control action.

Fig 5 further clarifies the internal mechanism of the proposed law. The residual activation surface $\chi_{i}$ becomes significant when the physical rate is small while the sliding variable remains nonzero. As a result, the effective reaching gain is locally increased in the residual region, instead of being enlarged over the entire operating domain. The reaching-rate surface shows that this additional action has the correct sign with respect to s, which preserves the sliding condition while avoiding discontinuous switching around s=0. This behavior is consistent with the Lyapunov-based interpretation in the previous subsection: the proposed law strengthens the descent rate only when residual motion is detected.

**Fig 5:**
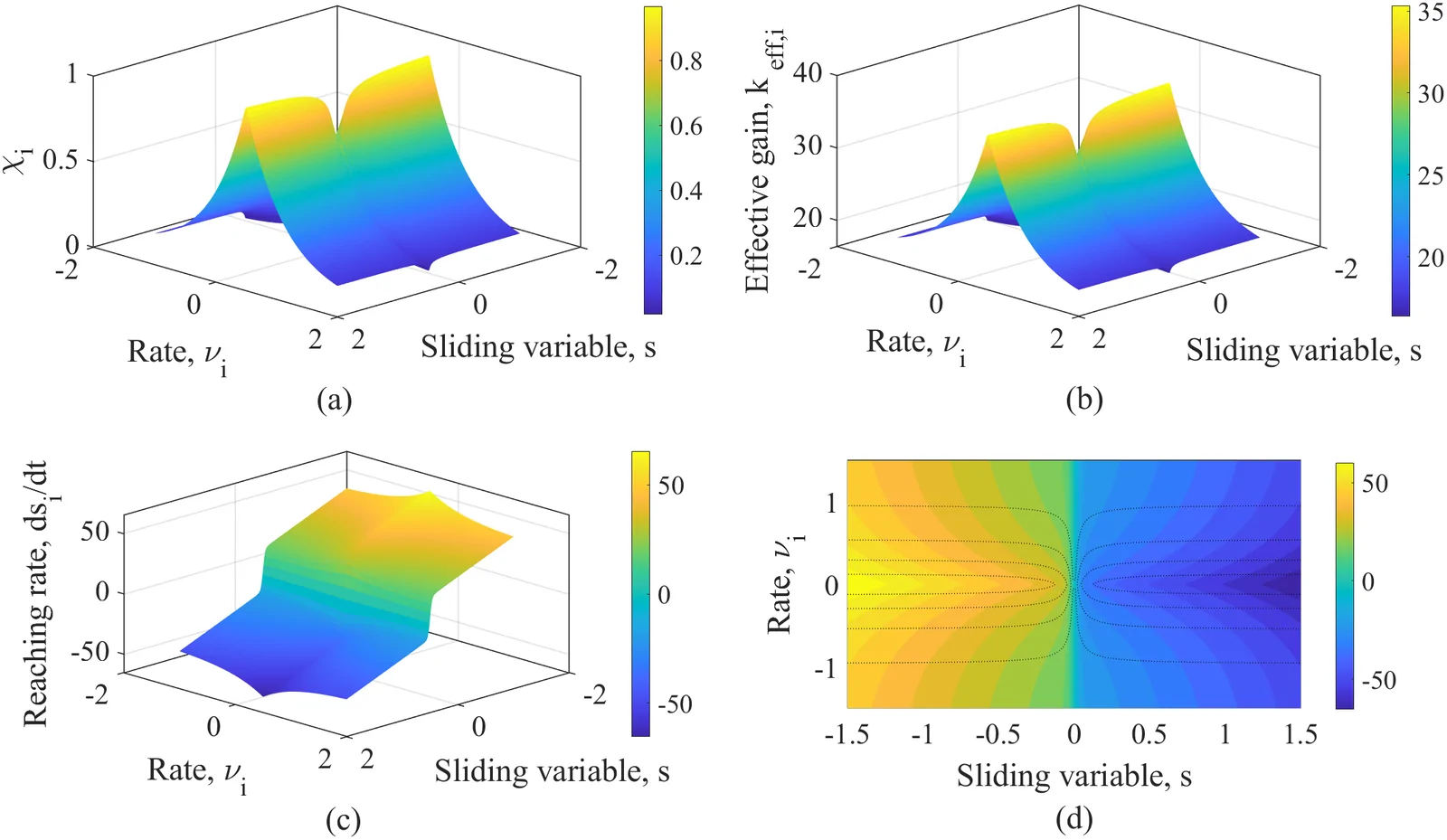
Residual activation and reaching-rate surfaces of the proposed FRMRL: (a) residual activation factor $\chi_{i}$, (b) effective reaching gain, (c) reaching-rate surface $\dot{s}$, and (d) contour interpretation in the $\left(s,\nu_{i}\right)$ plane.

To examine robustness against model-gain uncertainty, the plant gain is varied by the scale factors 0.75, 0.85, 1.00, 1.15, and 1.25, while the controller parameters are kept unchanged. Fig 6 shows that the proposed FRMRL maintains the smallest average IAE, the lowest average high-frequency control RMS, and the best normalized robustness score over the tested gain range. The average robustness score is 0.1387 for the proposed FRMRL, compared with 0.3681 for ERL and 0.7999 for CRL. This result indicates that the proposed residual-rate modulation improves robustness without relying on a large global switching gain.

**Fig 6:**
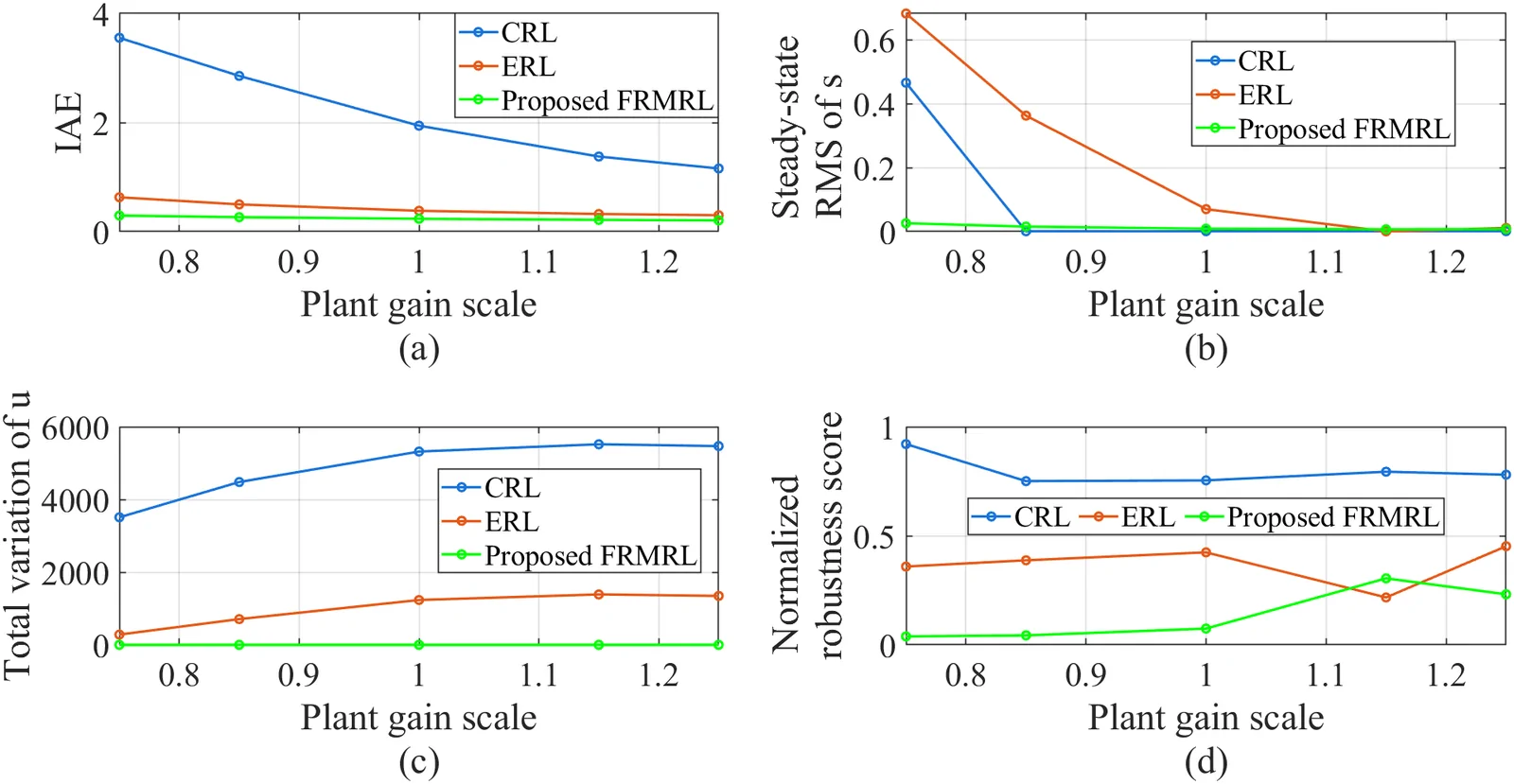
Robustness comparison under plant-gain mismatch: (a) IAE, (b) steady-state RMS of the sliding variable, (c) total variation of the control input, and (d) normalized robustness score under different plant-gain scales.

Overall, the numerical assessment shows that the proposed FRMRL does not merely smooth the conventional sign-based reaching law. Instead, it introduces a state-dependent residual mechanism that selectively reinforces the reaching action when low-rate residual error persists. This property explains why the proposed law achieves faster residual suppression and smoother control activity than CRL and ERL, and it provides the control-oriented basis for embedding the FRMRL into the quadrotor attitude–altitude controller in the next subsection.

### 3.4. Quadrotor controller and stability analysis

From (14) and $s_{i}={\dot{e}}_{i}+\lambda_{i}e_{i}$, the sliding-variable derivative is


$$\begin{array}{c} {\dot{s}}_{i}=f_{i}(X)+g_{i}(X)u_{i}+\Delta_{i}(t)-{\ddot{y}}_{i,d}+\lambda_{i}{\dot{e}}_{i}. \end{array}$$ (25)

To impose the proposed reaching dynamics, choose


$$\begin{array}{c} u_{i}=\frac{\text{1}}{g_{i}(X)}\left[-f_{i}(X)+{\ddot{y}}_{i,d}-\lambda_{i}{\dot{e}}_{i}-k_{s,i}s_{i}-\left(k_{c,i}+k_{r,i}\chi_{i}\right)\Phi_{i}\left(s_{i}\right)\right]. \end{array}$$ (26)

Substitution of (26) into (25) gives the closed-loop sliding dynamics


$$\begin{array}{c} {\dot{s}}_{i}=-k_{s,i}s_{i}-\left(k_{c,i}+k_{r,i}\chi_{i}\right)\Phi_{i}\left(s_{i}\right)+\Delta_{i}(t). \end{array}$$ (27)

For the quadrotor model, the corresponding virtual control inputs are


$$\begin{array}{c} U_{\text{2}}=I_{x}\left[-f_{\phi }+{\ddot{\phi }}_{d}-\lambda_{\phi }{\dot{e}}_{\phi }-k_{s,\phi }s_{\phi }-\left(k_{c,\phi }+k_{r,\phi }\chi_{\phi }\right)\text{tanh}\left(\frac{s_{\phi }}{\delta_{\phi }}\right)\right], \end{array}$$ (28)

$$\begin{array}{c} U_{\text{3}}=I_{y}\left[-f_{\theta }+{\ddot{\theta }}_{d}-\lambda_{\theta }{\dot{e}}_{\theta }-k_{s,\theta }s_{\theta }-\left(k_{c,\theta }+k_{r,\theta }\chi_{\theta }\right)\text{tanh}\left(\frac{s_{\theta }}{\delta_{\theta }}\right)\right], \end{array}$$ (29)

$$\begin{array}{c} U_{\text{4}}=I_{z}\left[-f_{\psi }+{\ddot{\psi }}_{d}-\lambda_{\psi }{\dot{e}}_{\psi }-k_{s,\psi }s_{\psi }-\left(k_{c,\psi }+k_{r,\psi }\chi_{\psi }\right)\text{tanh}\left(\frac{s_{\psi }}{\delta_{\psi }}\right)\right], \end{array}$$ (30)

$$\begin{array}{c} U_{\text{1}}=\frac{m}{\text{cos}\phi \text{cos}\theta }\left[g+{\ddot{z}}_{d}-\lambda_{z}{\dot{e}}_{z}-k_{s,z}s_{z}-\left(k_{c,z}+k_{r,z}\chi_{z}\right)\text{tanh}\left(\frac{s_{z}}{\delta_{z}}\right)\right]. \end{array}$$ (31)

The terms $f_{\phi }$, $f_{\theta }$, and $f_{\psi }$ are the nonlinear drift terms defined in Section 2. The altitude input is well defined because cosϕcosθ is strictly positive under the admissible attitude range.

**Remark 4.** The controller is implemented at the virtual-input level. The motor mixer converts $U_{\text{1}}$, $U_{\text{2}}$, $U_{\text{3}}$, and $U_{\text{4}}$ into rotor-speed commands. Any mismatch caused by motor dynamics, saturation, dead-zone, rotor drag, or partial motor effectiveness loss is included in the matched perturbation $\Delta_{i}(t)$. This treatment keeps the stability proof concise while still allowing realistic actuator nonlinearities to be included in the numerical validation.

Because the activation factor in Eq. (19) enters Eq. (20) algebraically, differentiating the Lyapunov function used in Theorem 1 gives Eq. (33) without a derivative of that factor. The resulting uniform ultimate boundedness estimate is independent of the rate at which the activation factor varies, provided the stated matched-perturbation bound holds uniformly and the smooth robust gain exceeds that bound. The first-order motor lag and rotor-level dead-zone in Eq. (5) are not explicitly compensated by the virtual-input controller; their net effect is included in the matched perturbation. Motor phase lag can delay or attenuate correction near the smooth boundary layer, so the theorem applies only while that perturbation bound remains valid. The simulations cover the specified actuator settings and do not establish stability for arbitrary motor lag or phase delay.

The stability result is now established for the complete closed-loop attitude–altitude subsystem.

Theorem 1. Consider the quadrotor attitude–altitude subsystem (14) under Assumptions 1–4. Let the sliding variable be defined by $s_{i}={\dot{e}}_{i}+\lambda_{i}e_{i}$, with $\lambda_{i}>\text{0}$, and let the control input be given by (26). If the lumped perturbation satisfies $\left|\Delta_{i}(t)\right|\leq {\bar{\Delta }}_{i}$ and $k_{c,i}>{\bar{\Delta }}_{i}$, then $s_{i}$ and $e_{i}$ are uniformly ultimately bounded. In the absence of lumped perturbations, both $s_{i}$ and $e_{i}$ converge to zero asymptotically. Moreover, in the low-rate residual regime, the proposed law provides a larger robust reaching margin than the smooth reaching law in (17).

**Proof.** Choose the Lyapunov function


$$\begin{array}{c} V_{i}=\frac{\text{1}}{\text{2}}s_{i}^{\text{2}}. \end{array}$$ (32)

Using (27), its time derivative is


$$\begin{array}{c} {\dot{V}}_{i}=-k_{s,i}s_{i}^{\text{2}}-\left(k_{c,i}+k_{r,i}\chi_{i}\right)s_{i}\Phi_{i}\left(s_{i}\right)+s_{i}\Delta_{i}(t). \end{array}$$ (33)

Since $s_{i}\Phi_{i}\left(s_{i}\right)\geq \text{0}$ and $\left|\Delta_{i}(t)\right|\leq {\bar{\Delta }}_{i}$,


$$\begin{array}{c} {\dot{V}}_{i}\leq -k_{s,i}s_{i}^{\text{2}}-\left(k_{c,i}+k_{r,i}\chi_{i}\right)\left|s_{i}\right|\left|\text{tanh}\left(\frac{s_{i}}{\delta_{i}}\right)\right|+{\bar{\Delta }}_{i}\left|s_{i}\right|. \end{array}$$ (34)

Let $\epsilon_{i}\in \left({\bar{\Delta }}_{i}/k_{c,i},\text{1}\right)$. Whenever


$$\begin{array}{c} \left|s_{i}\right|\geq \delta_{i}\text{arctanh}\left(\epsilon_{i}\right), \end{array}$$ (35)

one has $\left|\text{tanh}\left(s_{i}/\delta_{i}\right)\right|\geq \epsilon_{i}$. Therefore,


$$\begin{array}{c} {\dot{V}}_{i}\leq -k_{s,i}s_{i}^{\text{2}}-\left(k_{c,i}\epsilon_{i}-{\bar{\Delta }}_{i}\right)\left|s_{i}\right|<\text{0} \end{array}$$ (36)

outside the compact set


$$\begin{array}{c} \Omega_{s,i}=\left\{s_{i}:\left|s_{i}\right|<\delta_{i}\text{arctanh}\left(\epsilon_{i}\right)\right\}. \end{array}$$ (37)

Thus, $s_{i}$ is uniformly ultimately bounded. Since


$$\begin{array}{c} {\dot{e}}_{i}=-\lambda_{i}e_{i}+s_{i}, \end{array}$$ (38)

the tracking error is the response of an exponentially stable first-order system driven by the bounded signal $s_{i}$. Hence, $e_{i}$ is uniformly ultimately bounded and satisfies


$$\begin{array}{c} \underset{t\to \infty }{\text{limsup}}\left|e_{i}(t)\right|\leq \frac{\text{1}}{\lambda_{i}}\underset{t\to \infty }{\text{limsup}}\left|s_{i}(t)\right|. \end{array}$$ (39)

If $\Delta_{i}(t)=\text{0}$, (33) becomes


$$\begin{array}{c} {\dot{V}}_{i}=-k_{s,i}s_{i}^{\text{2}}-\left(k_{c,i}+k_{r,i}\chi_{i}\right)s_{i}\Phi_{i}\left(s_{i}\right)\leq -k_{s,i}s_{i}^{\text{2}}=-\text{2}k_{s,i}V_{i}. \end{array}$$ (40)

Therefore, $s_{i}$ converges exponentially to zero. From ${\dot{e}}_{i}+\lambda_{i}e_{i}=s_{i}$, it follows that $e_{i}\to \text{0}$.

It remains to show the stronger residual-mode robustness. In the low-rate residual regime, Lemma 1 gives $\chi_{i}\geq {\underset{―}{\chi }}_{i}$. Hence, the effective smooth reaching gain satisfies


$$\begin{array}{c} k_{c,i}+k_{r,i}\chi_{i}\geq k_{c,i}+k_{r,i}{\underset{―}{\chi }}_{i}. \end{array}$$ (41)

Compared with the smooth law in (17), whose robust smooth gain is only $k_{c,i}$, the proposed law increases the local robust margin by at least $k_{r,i}{\underset{―}{\chi }}_{i}$. Consequently, in the residual regime, the Lyapunov descent satisfies


$$\begin{array}{c} {\dot{V}}_{i}\leq -k_{s,i}s_{i}^{\text{2}}-\left[\left(k_{c,i}+k_{r,i}{\underset{―}{\chi }}_{i}\right)\left|\text{tanh}\left(\frac{s_{i}}{\delta_{i}}\right)\right|-{\bar{\Delta }}_{i}\right]\left|s_{i}\right|. \end{array}$$ (42)

This proves that the proposed reaching law provides a stronger disturbance-rejection margin than the conventional smooth reaching law whenever the system operates in the low-rate residual regime.

**Remark 5.** The theorem establishes practical stability under bounded perturbations and asymptotic convergence in the nominal case. This is the expected stability property of a smooth sliding-mode controller. Exact finite-time convergence cannot be claimed for the smooth law because $\text{tanh}\left(s_{i}/\delta_{i}\right)$ removes the discontinuity at the origin. The advantage of the proposed law is not exact finite-time reaching, but improved residual-error suppression with bounded and smooth control action.

Accordingly, the UUB result is conditional on the actuator-induced mismatch remaining a matched perturbation that satisfies Theorem 1, including $\Delta_{\text{i}}^{¯}\;<\;{\text{k}}_{\text{c},\text{i}}$. If saturation or loss of effectiveness makes the requested virtual input unattainable and this bound is violated, the theorem does not establish UUB. Scenario S6 tests only the specified partial reduction to η = [1, 0.68, 1, 0.82] from t = 6 s; it does not establish robustness to complete rotor failure or arbitrary loss of control authority.

In the complete cascade architecture, the outer position loop computes $a_{x}^{d}$, $a_{y}^{d}$, and $a_{z}^{d}$, which are converted into $\phi_{d}$, $\theta_{d}$, and $T_{d}$. The inner loop then applies (28)–(31) to generate the virtual inputs $U_{\text{1}}$, $U_{\text{2}}$, $U_{\text{3}}$, and $U_{\text{4}}$. These inputs are mapped to rotor-speed commands by the motor mixer and then passed through the actuator saturation and motor dynamics.

The proposed controller therefore replaces only the inner attitude–altitude stabilizing block. This preserves the standard cascade structure used in quadrotor flight control while allowing the proposed reaching law to be evaluated fairly against PID, sign-based SMC, smooth SMC, adaptive SMC, and finite-time or higher-order SMC benchmarks.

## 4. Results and discussion

This section evaluates the proposed FRMRL-based attitude–altitude controller under six representative UAV operating scenarios: nominal tracking, constant matched disturbance, time-varying wind-like disturbance, parametric uncertainty, actuator saturation with dead-zone, and partial motor effectiveness loss. The main plotted comparison includes PID, CRL-SMC, ERL-SMC, ASMC, and the proposed FRMRL. In addition, SERL-SMC is retained in the quantitative assessment as an ablation baseline. This baseline uses the same smooth reaching gains $k_{s}$, $k_{c}$, and δ as the proposed FRMRL, but excludes the residual activation term $k_{r}\chi_{i}$. Therefore, the SERL-SMC comparison isolates the contribution of the proposed residual-mode correction. All methods are evaluated under the same reference trajectories, nominal controller model, actuator limits, and disturbance profiles. The nominal UAV parameters are m=0.65 kg, $I_{x}=I_{y}=\text{7}.\text{5}\times {\text{10}}^{-\text{3}}$ kg m², $I_{z}=\text{1}.\text{3}\times {\text{10}}^{-\text{2}}$ kg m², l=0.23 m, $b=\text{3}.\text{13}\times {\text{10}}^{-\text{5}}$ N/(rad/s)², $d=\text{7}.\text{50}\times {\text{10}}^{-\text{7}}$ N m/(rad/s)², and $\tau_{m}=\text{0}.\text{025}$ s. The sliding-surface gains are selected as $\lambda =[\text{8},\text{8},\text{5}.\text{5},\text{4}.\text{5}{]}^{T}$ for the roll, pitch, yaw, and altitude channels, respectively. For the proposed FRMRL, the smooth base gains are $k_{s}=[\text{5}.\text{2},\text{5}.\text{2},\text{3}.\text{7},\text{4}.\text{3}{]}^{T}$, $k_{c}=[\text{3}.\text{1},\text{3}.\text{1},\text{2}.\text{1},\text{3}.\text{5}{]}^{T}$, and $\delta =[\text{0}.\text{035},\text{0}.\text{035},\text{0}.\text{045},\text{0}.\text{055}{]}^{T}$, whereas the residual-mode gains are $k_{r}=[\text{4}.\text{6},\text{4}.\text{6},\text{3}.\text{0},\text{4}.\text{4}{]}^{T}$, $v_{\text{rate}}=[\text{0}.\text{22},\text{0}.\text{22},\text{0}.\text{18},\text{0}.\text{30}{]}^{T}$, and $\sigma =[\text{0}.\text{025},\text{0}.\text{025},\text{0}.\text{035},\text{0}.\text{045}{]}^{T}$.

The performance is assessed using total IAE, steady-state IAE over 5–15 s, peak tracking error, maximum overshoot in the first 5 s, settling time, control total variation, high-frequency control RMS, steady-state control ripple, and a journal-oriented quality score. Lower values indicate better performance for these indices. The journal-oriented score is an author-selected, dimensionless summary of normalized measures and is reported only as a secondary descriptor; the individual metrics are the primary basis for comparison. For clarity, the six benchmark scenarios are denoted consistently throughout this section. S1 corresponds to nominal attitude–altitude tracking; S2 introduces a constant matched disturbance; S3 applies a time-varying wind-like disturbance; S4 evaluates parameter mismatch between the controller model and the plant; S5 combines actuator saturation with rotor-speed dead-zone effects; and S6 represents partial motor-effectiveness loss, which is interpreted as reduced actuation authority rather than complete rotor failure. This notation is used in the tracking-error responses, control-input comparisons, residual-activation analysis, and cross-scenario quantitative assessment. $\text{J }=\;\text{0}.\text{14 }{\text{n}}_{\text{T}}\;+\;\text{0}.\text{12 }{\text{n}}_{\text{O}}\;+\;\text{0}.\text{18 }{\text{n}}_{\text{P}}\;+\;\text{0}.\text{22 }{\text{n}}_{\text{IAE},\text{ss}}\;+\;\text{0}.\text{14 }{\text{n}}_{\text{MAE},\text{ss}}\;+\;\text{0}.\text{14 }{\text{n}}_{\text{R}\_\text{y}}\;+\;\text{0}.\text{06 }{\text{n}}_{\text{R}\_\text{u}}$; ${\text{n}}_{\text{X},\text{s},\text{j}}\;=\;{\text{X}}_{\text{s},\text{j}}\;/\;{\text{max}}_{\text{k}\in \text{C}}{\text{X}}_{\text{s},\text{k}}$. Here, *T* is all-channel settling time; *O* and *P* are maximum overshoot and peak absolute tracking error over 0–5 s; IAE_ss_ and MAE_ss_ are steady-state error measures over 5–15 s; and *R*_y_ and *R*_u_ are output-ripple and control-ripple measures over 5–15 s. Each metric is normalized by its maximum across the six controllers in the same scenario. If a controller has infinite all-channel settling time, it is assigned 1.20 times the largest finite value in that scenario before normalization; if no finite settling time is available, the replacement value is 1. The weights assign 44% to transient response, 50% to steady-state tracking and output ripple, and 6% to control ripple. They were selected to balance transient and steady-state behavior while giving control activity a smaller supporting weight; they are not empirically calibrated. Thus, *J* is a study-specific relative index, not a physical quantity, and its ranking depends on the selected metrics and weights. The scenario-by-scenario discussion below prioritizes these individual measures and uses the score only as a supplementary summary.

The channel disturbance used to define the six scenarios is


$$\begin{array}{c} \Delta_{\text{i}}(\text{t})\;=\;{\text{d}}_{\text{c},\text{i}}\;+\;{\text{w}}_{\text{i}}(\text{t})\;+\;{\text{A}}_{\text{r},\text{i}}\text{ exp}(-|{\dot{\text{y}}}_{\text{i}}|/{\text{v}}_{\text{r},\text{i}})\text{ tanh}({\text{s}}_{\text{i}}/\delta_{\text{r},\text{i}}) \end{array}$$

The vectors are ordered [roll, pitch, yaw, altitude]; the first three disturbance components are in rad s^−2^ and the altitude component is in m s^−2^. Here, *d*_c_ is the constant disturbance, *w* is the wind-like term (zero except in S3), and *A*_r_ is the rate-gated residual amplitude (zero except in S5 and S6). When active, *v*_r_=[0.20, 0.20, 0.15, 0.25] and *δ*_r_=[0.050, 0.050, 0.060, 0.080]. Plant-scale vectors multiply the nominal values listed above; effectiveness vectors use rotor order [1, 2, 3, 4]. Unless a row states otherwise, actuator settings are *τ*_m_ = 0.025 s, *Ω*_min_ = 0, *Ω*_max_ = 850 rad s^−1^, *Ω*_dz_ = 0, and *η*=[1, 1, 1, 1]. Before motor allocation, virtual inputs are clipped to 0 ≤ *U*_1_ ≤ 2.4*m*_0_*g*, |*U*_2_| ≤ 2.0 N m, |*U*_3_| ≤ 2.0 N m, and |*U*_4_| ≤ 1.2 N m. The disturbance profiles, plant variations, and actuator settings for the six scenarios are summarized in Table 1.

**Table 1:** Exact disturbance, plant, and actuator settings for Scenarios S1–S6.

Scenario	Exact disturbance, plant, and actuator settings
**S1**	$d_{\text{c}}=[\text{0},\text{0},\text{0},\text{0}]$; $A_{\text{w}}=A_{\text{r}}=\text{0}$. Plant scales $\left[m,I_{\text{x},}\;I_{\text{y},}\;I_{\text{z},}\;b,d]=[\text{1},\text{1},\text{1},\text{1},\text{1},\text{1}\right]$. $\tau_{\text{m}}=\text{0}.\text{025 s}$; $\Omega_{\text{max}}=\text{850 rad}/\text{s}$; $\Omega_{\text{dz}}=\text{0}$; η=[1,1,1,1].
**S2**	$d_{\text{c}}=[\text{0}.\text{35},-\text{0}.\text{30},\text{0}.\text{22},\text{0}.\text{28}]$; $A_{\text{w}}=A_{\text{r}}=\text{0}$. Plant scales [1,1,1,1,1,1]. $\tau_{\text{m}}=\text{0}.\text{025 s}$; $\Omega_{\text{max}}=\text{850 rad}/\text{s}$; $\Omega_{\text{dz}}=\text{0}$; η=[1,1,1,1].
**S3**	$d_{\text{c}}=\text{0}$; $A_{\text{w}}=[\text{0}.\text{30},\text{0}.\text{28},\text{0}.\text{18},\text{0}.\text{35}]$; $A_{\text{r}}=\text{0}$; wind and gust terms are specified below. Plant scales [1,1,1,1,1,1]. $\tau_{\text{m}}=\text{0}.\text{025 s}$; $\Omega_{\text{max}}=\text{850 rad}/\text{s}$; $\Omega_{\text{dz}}=\text{0}$; η=[1,1,1,1].
**S4**	$d_{\text{c}}=[\text{0}.\text{10},-\text{0}.\text{10},\text{0}.\text{08},\text{0}.\text{12}]$; $A_{\text{w}}=A_{\text{r}}=\text{0}$. Plant scales $\left[m,I_{\text{x},}\;I_{\text{y},}\;I_{\text{z},}\;b,d]=[\text{1}.\text{15},\text{1}.\text{25},\text{0}.\text{82},\text{1}.\text{18},\text{0}.\text{90},\text{1}.\text{20}\right]$. $\tau_{\text{m}}=\text{0}.\text{025 s}$; $\Omega_{\text{max}}=\text{850 rad}/\text{s}$; $\Omega_{\text{dz}}=\text{0}$; η=[1,1,1,1].
**S5**	$d_{\text{c}}=A_{\text{w}}=\text{0}$; $A_{\text{r}}=[\text{0}.\text{22},-\text{0}.\text{20},\text{0}.\text{15},\text{0}.\text{18}]$. Plant scales [1,1,1,1,1,1]. $\tau_{\text{m}}=\text{0}.\text{055 s}$; $\Omega_{\text{max}}=\text{520 rad}/\text{s}$; $\Omega_{\text{dz}}=\text{28 rad}/\text{s}$; η=[1,1,1,1].
**S6**	$d_{\text{c}}=[\text{0}.\text{10},-\text{0}.\text{08},\text{0}.\text{05},\text{0}.\text{08}]$; $A_{\text{w}}=\text{0}$; $A_{\text{r}}=[\text{0}.\text{15},-\text{0}.\text{12},\text{0}.\text{10},\text{0}.\text{12}]$. Plant scales [1,1,1,1,1,1]. At t=6 s, η changes from [1,1,1,1] to [1,0.68,1,0.82]. $\tau_{\text{m}}=\text{0}.\text{045 s}$; $\Omega_{\text{max}}=\text{850 rad}/\text{s}$; $\Omega_{\text{dz}}=\text{12 rad}/\text{s}$.

The S3 wind-like term is defined by


$$\begin{array}{c} \text{w}(\text{t})\;=\;{\text{A}}_{\text{w}}\;⊙\text{ f}(\text{t})\;+\text{ g}(\text{t}),\;{\text{A}}_{\text{w}}\;=[\text{0}.\text{30},\;\text{0}.\text{28},\;\text{0}.\text{18},\;\text{0}.\text{35}{]}^{T} \end{array}$$

$$\begin{array}{c} {\text{f}}_{\text{1}}(\text{t})\;=\text{ sin}(\text{1}.\text{20 t})\;+\;\text{0}.\text{35 sin}(\text{3}.\text{10 t }+\;\text{0}.\text{2}) \end{array}$$

$$\begin{array}{c} {\text{f}}_{\text{2}}(\text{t})\;=\text{ sin}(\text{1}.\text{05 t }+\;\text{0}.\text{6})\;+\;\text{0}.\text{30 sin}(\text{2}.\text{70 t}) \end{array}$$

$$\begin{array}{c} {\text{f}}_{\text{3}}(\text{t})\;=\text{ sin}(\text{0}.\text{80 t }+\;\text{0}.\text{4})\;+\;\text{0}.\text{25 sin}(\text{2}.\text{30 t }+\;\text{0}.\text{7}) \end{array}$$

$$\begin{array}{c} {\text{f}}_{\text{4}}(\text{t})\;=\text{ sin}(\text{0}.\text{65 t }+\;\text{0}.\text{3})\;+\;\text{0}.\text{25 sin}(\text{1}.\text{90 t}) \end{array}$$

$$\begin{array}{c} \text{g}(\text{t})\;=\text{ exp}[-{\left((\text{t }-\;\text{7}.\text{5})/\text{0}.\text{55}\right)}^{\text{2}}]\;[\text{0}.\text{35},\;-\text{0}.\text{30},\;\text{0}.\text{18},\;\text{0}.\text{45}{]}^{T} \end{array}$$

### 4.1. Tracking-error responses under six UAV scenarios

Fig 7 shows the nominal attitude–altitude tracking case. The proposed FRMRL yields a well-damped response in all four controlled channels and avoids the residual oscillation visible in the sign-based CRL-SMC. In this case, the proposed method achieves the lowest total IAE of 0.8134, compared with 0.8954 for SERL-SMC, 0.9103 for ERL-SMC, 1.0767 for ASMC, 1.2835 for CRL-SMC, and 5.8784 for PID. The all-channel settling time is approximately 1.254 s, and no overshoot is observed in the first 5 s. Relative to the SERL-SMC ablation, the proposed residual term reduces the full-run IAE by 9.16% while preserving essentially the same steady-state smoothness. This confirms that the residual activation improves the transient-to-residual transition without requiring a larger global smooth gain.

**Fig 7:**
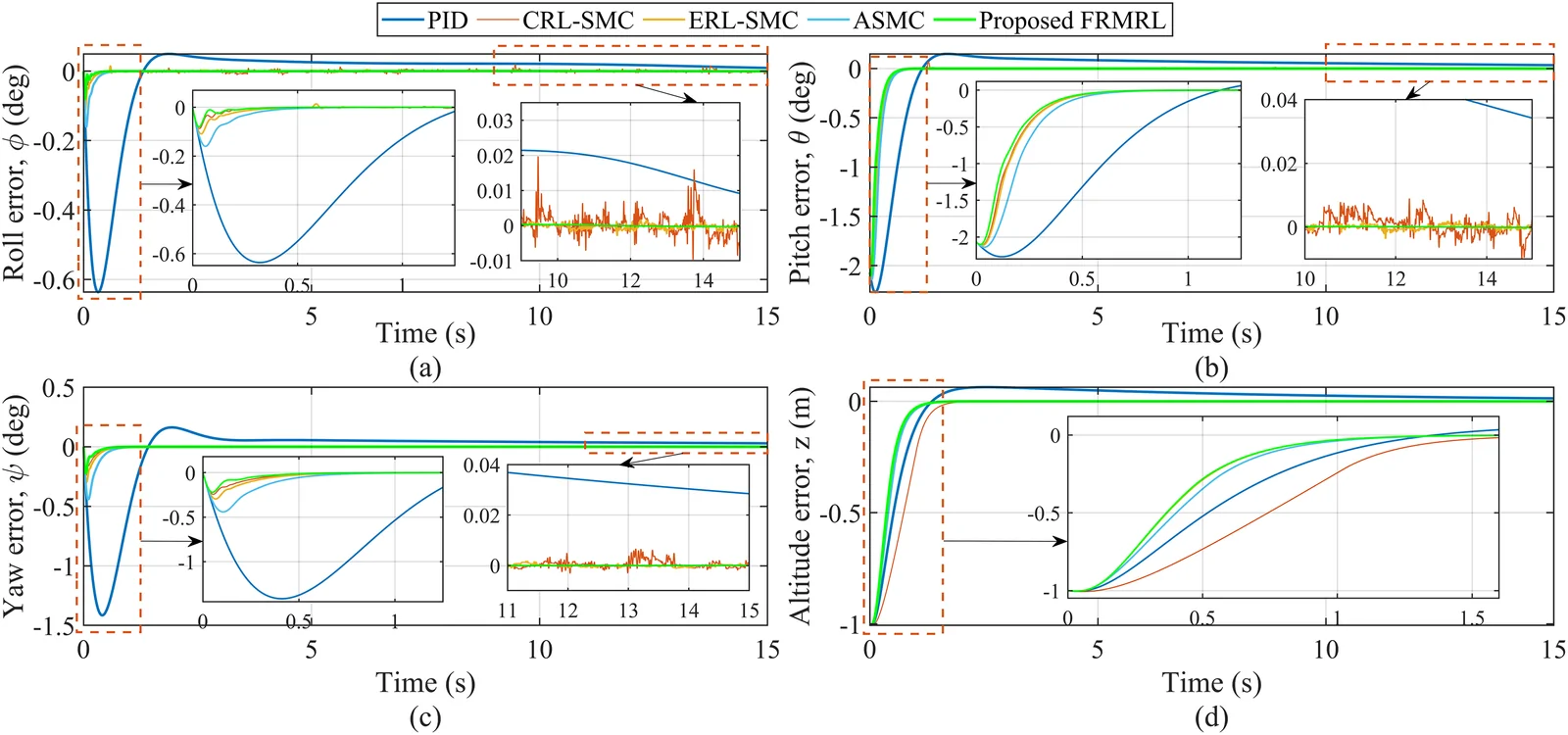
Tracking-error responses under UAV scenario S1: nominal attitude–altitude tracking: (a) roll error, (b) pitch error, (c) yaw error, and (d) altitude error.

Under constant matched disturbance, shown in Fig 8, PID develops large residual errors in the attitude channels, whereas the sliding-mode-based controllers maintain bounded tracking. ERL-SMC gives the smallest total IAE in this scenario, 0.9483, but it does so with a much larger control-ripple level than the smooth controllers. The proposed FRMRL gives a total IAE of 2.0421, which is lower than 2.2656 for SERL-SMC and 2.3982 for ASMC. The control ripple of FRMRL is 0.1170, whereas ERL-SMC reaches 1.5565. Thus, although ERL-SMC can reduce the integral tracking error in this biased case, the proposed FRMRL provides a more favorable balance between residual suppression and actuator smoothness.

**Fig 8:**
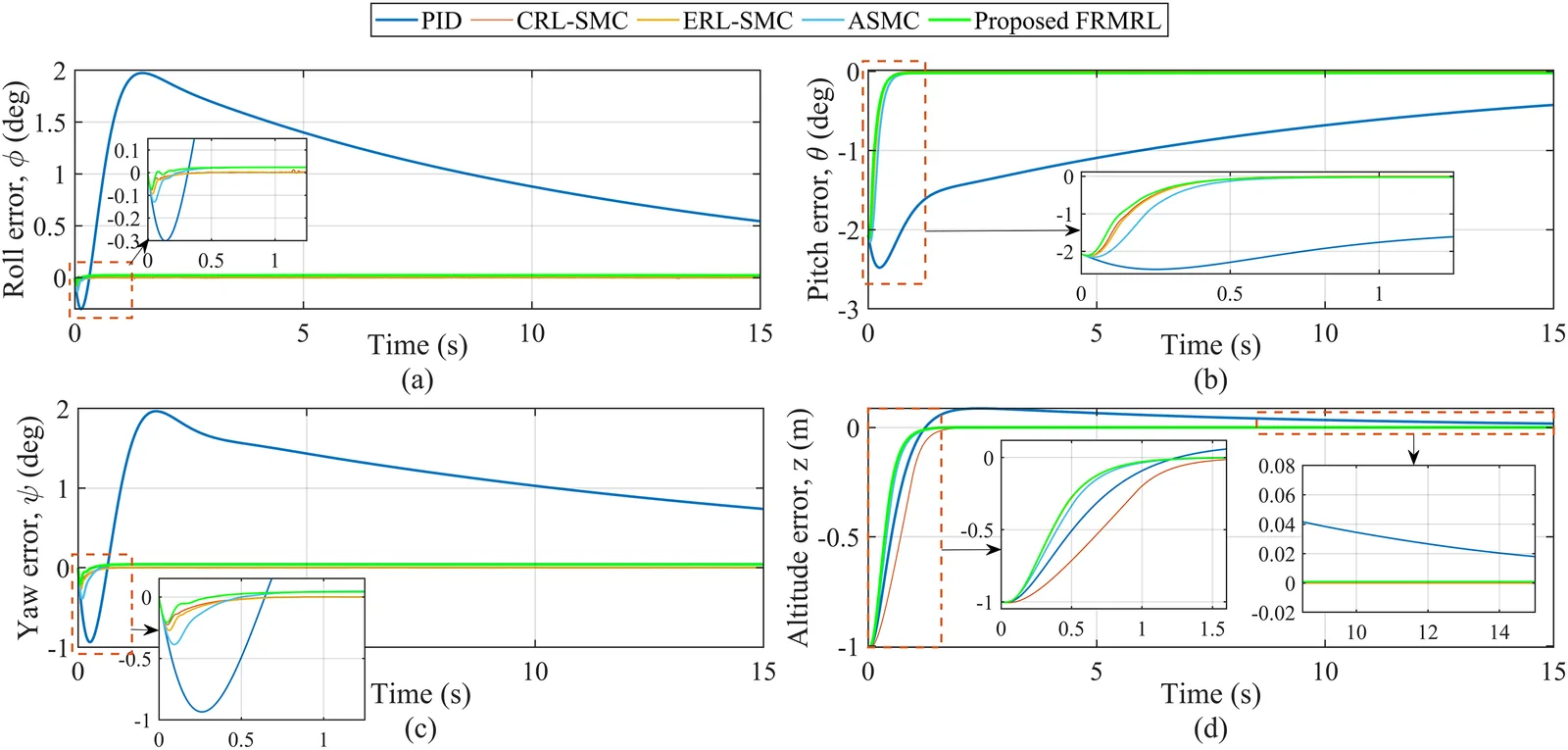
Tracking-error responses under UAV scenario S2: constant matched disturbance: (a) roll error, (b) pitch error, (c) yaw error, and (d) altitude error.

Fig 9 presents the time-varying wind-like disturbance case. This condition is more demanding because the perturbation is continuously varying rather than a fixed offset. PID shows pronounced oscillations in the roll and yaw channels, while CRL-SMC suppresses the error at the cost of high-frequency torque activity. ERL-SMC again gives a smaller total IAE, 0.9095, but with a control ripple of 1.6208. By contrast, the proposed FRMRL gives a total IAE of 1.5104, lower than 1.6456 for SERL-SMC and 1.7783 for ASMC, while keeping the control ripple at 0.4695. The residual activation term therefore improves the smooth-reaching baseline under wind-like perturbations without moving toward the high control variation associated with ERL-SMC.

**Fig 9:**
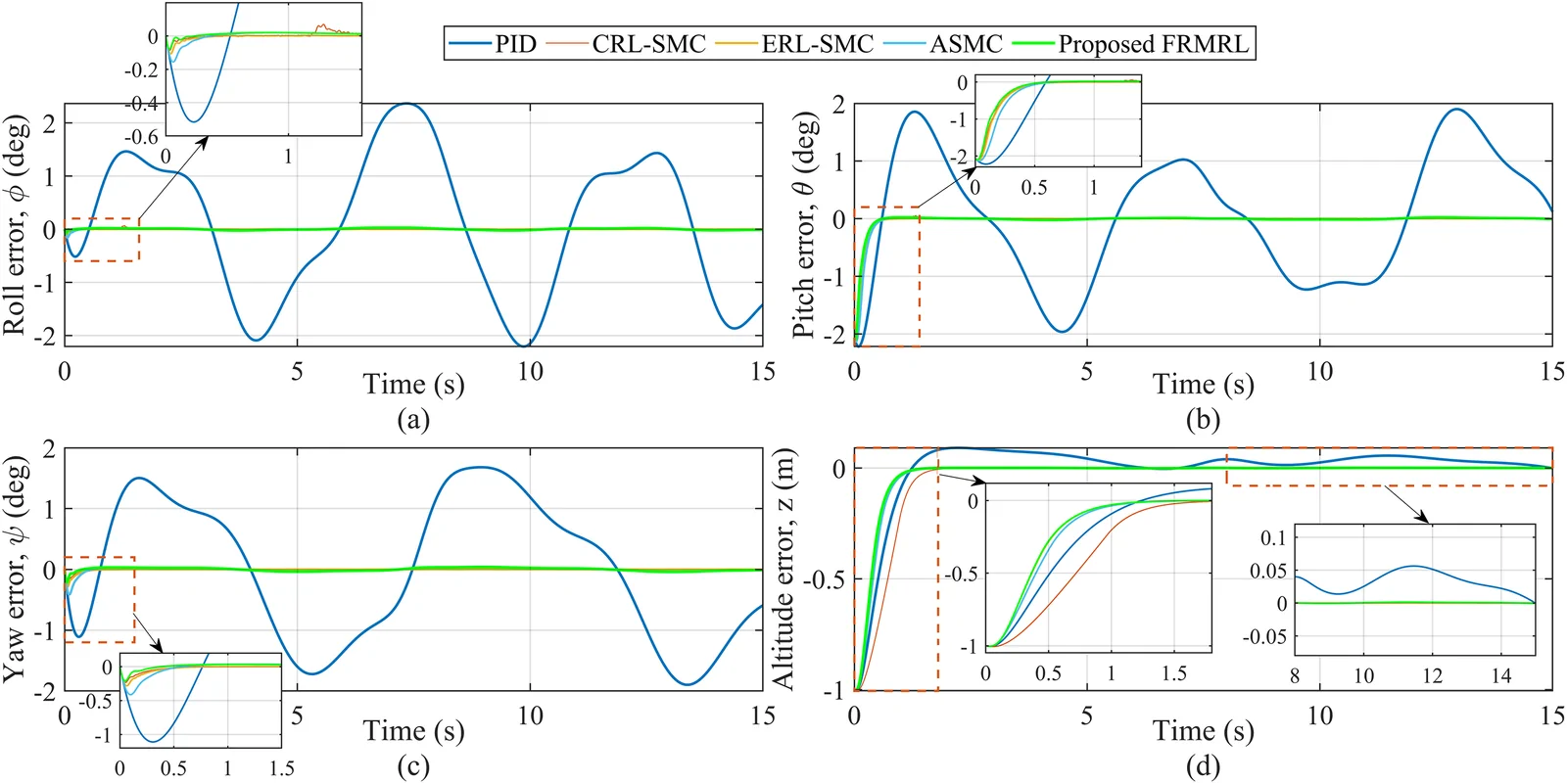
Tracking-error responses under UAV scenario S3: time-varying wind-like disturbance: (a) roll error, (b) pitch error, (c) yaw error, and (d) altitude error.

Fig 10 evaluates robustness against parametric uncertainty. In this scenario, the plant parameters are changed to $m=\text{1}.\text{15}m_{\text{0}}$, $I_{x}=\text{1}.\text{25}I_{x\text{0}}$, $I_{y}=\text{0}.\text{82}I_{y\text{0}}$, $I_{z}=\text{1}.\text{18}I_{z\text{0}}$, $b=\text{0}.\text{90}b_{\text{0}}$, and $d=\text{1}.\text{20}d_{\text{0}}$, while the controller keeps the nominal model. The proposed FRMRL maintains stable tracking with limited transient deviation. Its total IAE is 1.4425, lower than 1.5899 for SERL-SMC and 1.8379 for ASMC, although ERL-SMC gives a smaller IAE of 0.9977. The key distinction is actuator activity: the control ripple of ERL-SMC is 1.0051, whereas the proposed FRMRL keeps it at 0.1363. This result indicates that the proposed reaching law is robust to model-gain and inertia mismatch without relying on persistent switching activity.

**Fig 10:**
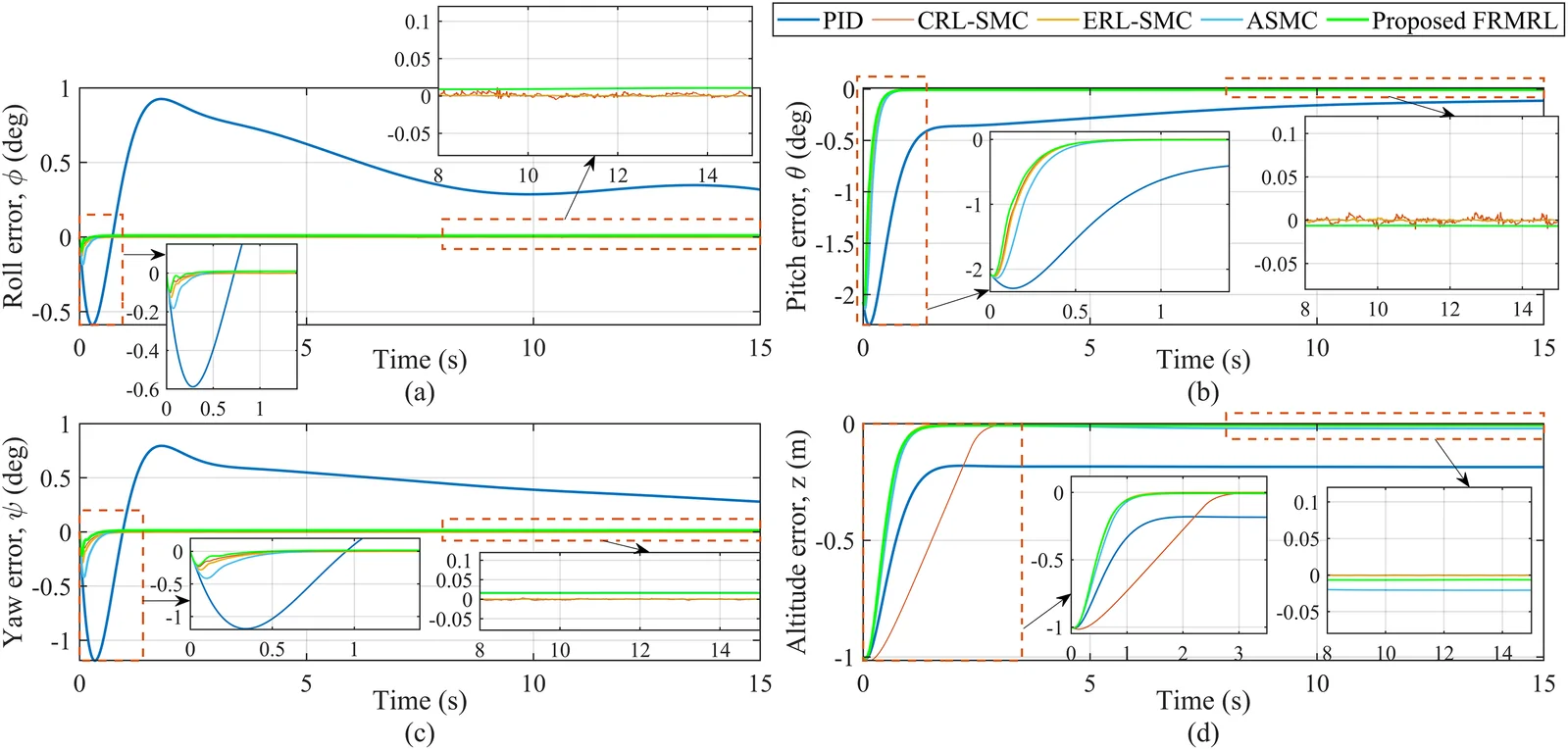
Tracking-error responses under UAV scenario S4: parameter uncertainty: (a) roll error, (b) pitch error, (c) yaw error, and (d) altitude error.

The actuator saturation and dead-zone case is shown in Fig 11. This scenario is particularly relevant to the proposed design because motor lag, rotor-speed limitation, and dead-zone effects create low-rate residual errors around the sliding surface. In this case, the proposed FRMRL has the lowest total IAE among the compared methods. Its total IAE is 1.0068, compared with 1.0641 for ERL-SMC, 1.1981 for SERL-SMC, 1.5663 for ASMC, and 2.1388 for CRL-SMC. The control ripple is only 0.1145, close to the smooth ablation baseline and far below 0.6972 for ERL-SMC. This result directly supports the intended role of the residual term: it compensates actuator-induced residual errors without reintroducing the high-frequency behavior of a sign-type reaching law.

**Fig 11:**
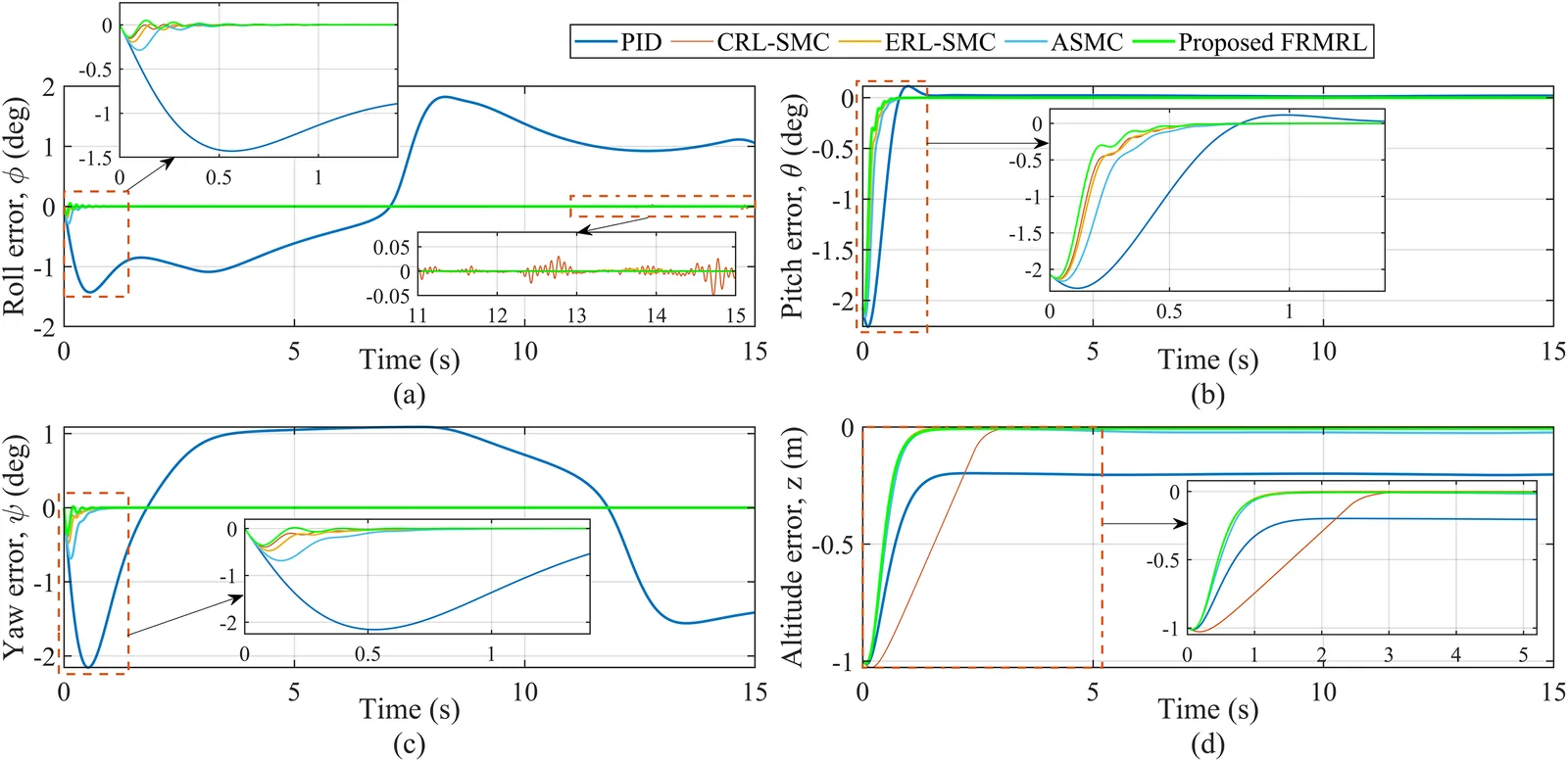
Tracking-error responses under UAV scenario S5: actuator saturation plus dead-zone: (a) roll error, (b) pitch error, (c) yaw error, and (d) altitude error.

Fig 12 considers partial motor effectiveness loss occurring at t=6 s. This case should be interpreted as partial loss of actuator effectiveness, not complete rotor failure. After the effectiveness reduction is introduced, PID and CRL-SMC exhibit severe degradation in at least one channel. ERL-SMC and ASMC remain more stable, but their residual errors and control ripples increase. The proposed FRMRL achieves the lowest IAE among the methods that maintain stable tracking, 86.4659, compared with 128.1774 for ERL-SMC, 134.6704 for SERL-SMC, and 170.5333 for ASMC. Its steady-state IAE is 85.5004, which is 32.79% lower than ERL-SMC and 36.00% lower than SERL-SMC. The proposed controller also yields the lowest steady-state control ripple, 1.3130, compared with 1.7258 for ERL-SMC, 1.5360 for SERL-SMC, and 1.7325 for ASMC. This case is the clearest evidence that the residual-mode correction becomes most valuable when the actuator authority is degraded and near-surface residual motion cannot be removed by a purely smooth reaching law alone.

**Fig 12:**
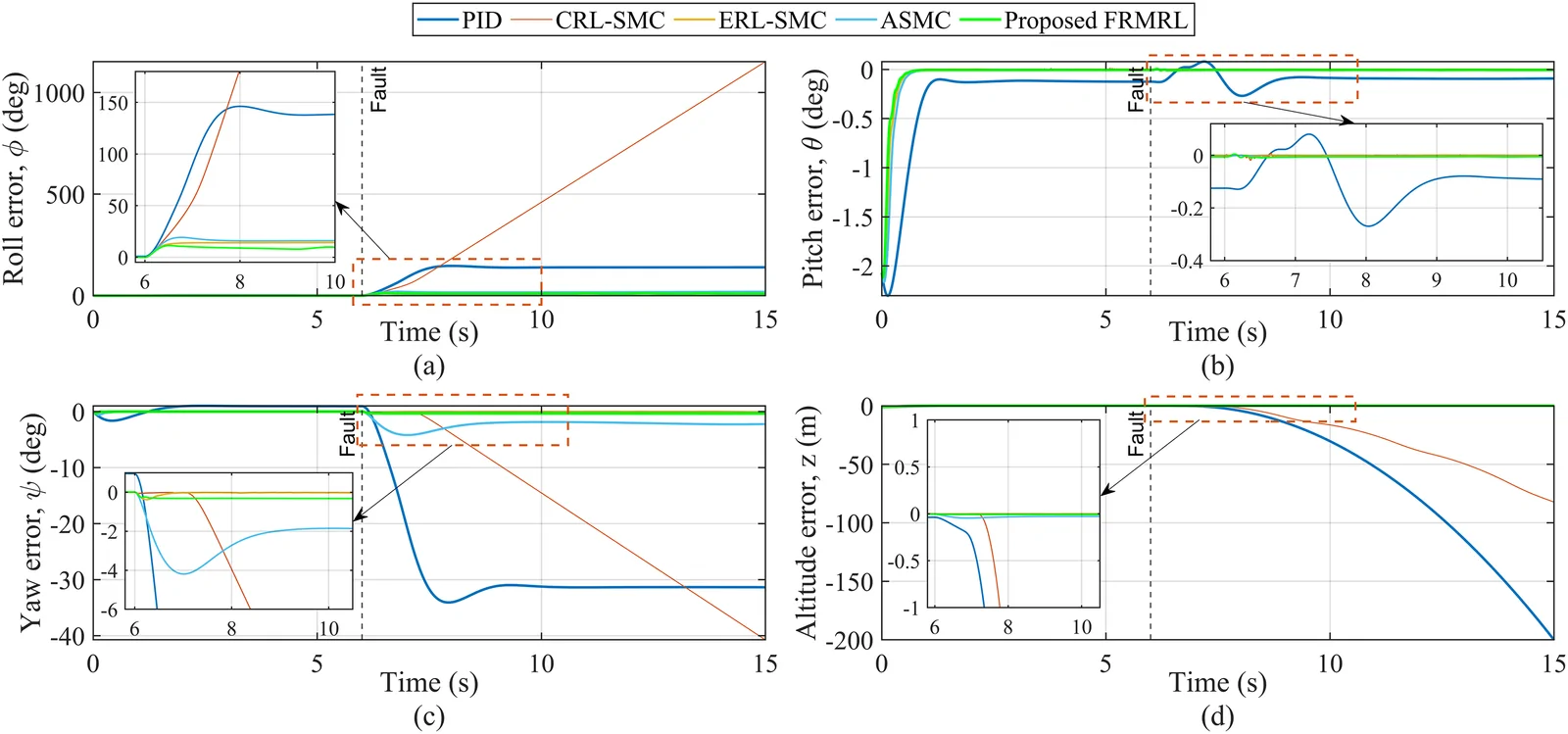
Tracking-error responses under UAV scenario S6: partial motor effectiveness loss: (a) roll error, (b) pitch error, (c) yaw error, and (d) altitude error.

### 4.2. Control-input activity

The control-input activities for Scenarios S1–S3 are shown in Fig 13. CRL-SMC generates visible high-frequency oscillations in the torque commands because of its discontinuous reaching term. ERL-SMC reduces part of this effect but still produces a relatively large control variation. In contrast, the proposed FRMRL keeps the thrust and torque commands close to those of the smooth controllers while improving the residual-error behavior. This distinction is important because a UAV controller should not be judged only by error reduction; excessive torque variation is directly relevant to actuator wear, power demand, and command feasibility.

**Fig 13:**
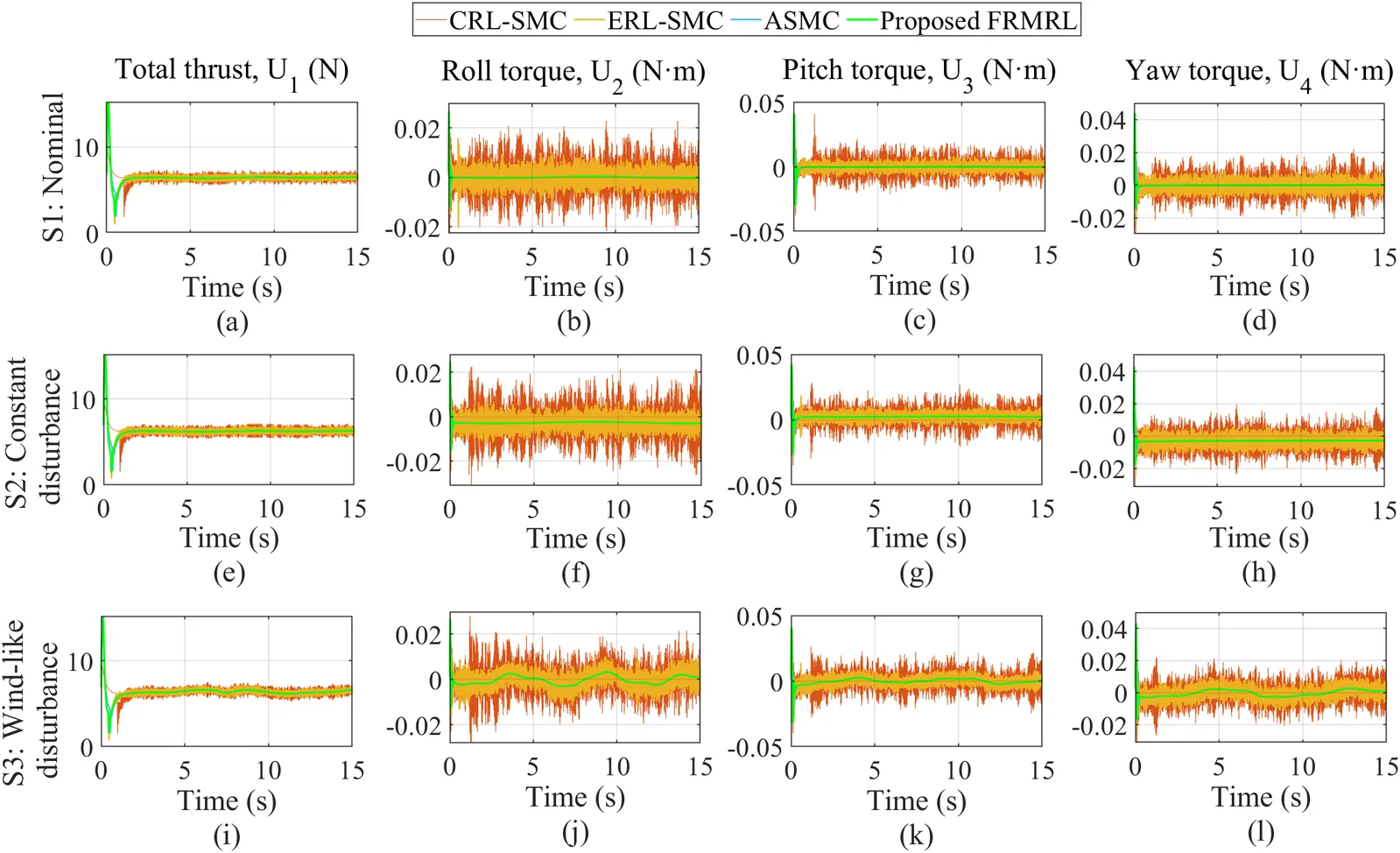
Control-input activities under UAV scenarios S1–S3: thrust and torque commands for (S1) nominal tracking, (S2) constant matched disturbance, and (S3) time-varying wind-like disturbance.

Averaged over all six scenarios, the proposed FRMRL reduces the control ripple peak-to-peak value to 0.3783, compared with 1.3285 for ERL-SMC and 2.6907 for CRL-SMC. The corresponding reductions are approximately 71.5% relative to ERL-SMC and 85.9% relative to CRL-SMC. Compared with ASMC, the proposed method reduces the average control ripple from 0.4487 to 0.3783. Its high-frequency control RMS is 0.0852, substantially lower than 0.1276 for ERL-SMC and 0.1448 for CRL-SMC, and close to the smooth ablation level. These results show that the residual term improves tracking robustness without pushing the controller toward a discontinuous switching regime.

Fig 14 presents the control-input activities for Scenarios S4–S6. Under parameter uncertainty and actuator nonlinearities, the proposed method maintains bounded and smooth control effort. Under partial motor effectiveness loss, the required control activity increases because the controller must compensate for reduced actuator authority. Nevertheless, the proposed FRMRL gives the smallest steady-state control ripple in Scenario S6, 1.3130, compared with 1.7258 for ERL-SMC, 1.5360 for SERL-SMC, 1.7325 for ASMC, and 6.8716 for CRL-SMC. The grouped control-input plots therefore confirm the main design premise: the proposed residual activation strengthens near-surface correction only when needed, rather than increasing switching intensity throughout the response.

**Fig 14:**
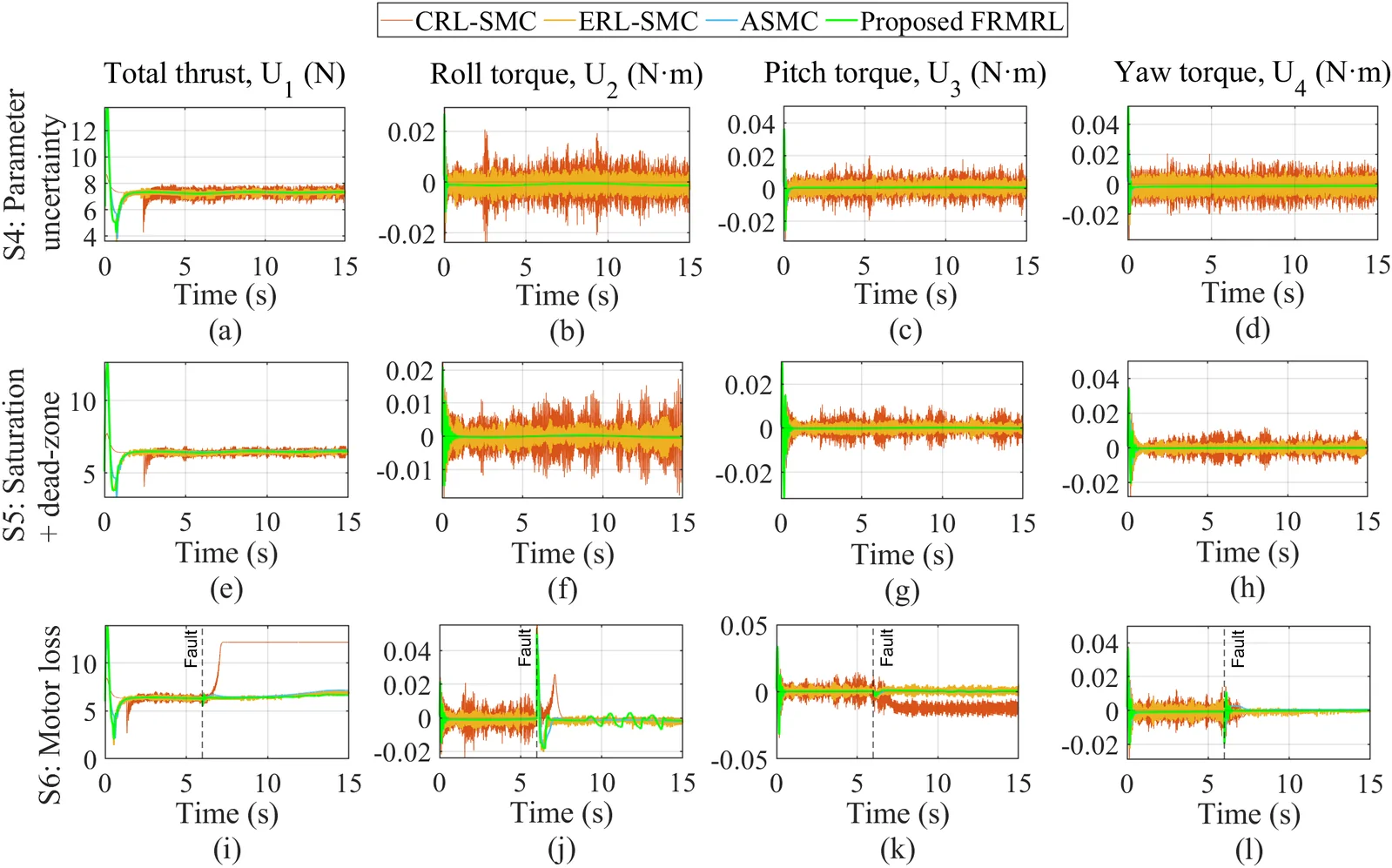
Control-input activities under UAV scenarios S4–S6: thrust and torque commands for (S4) parameter uncertainty, (S5) actuator saturation plus dead-zone, and (S6) partial motor effectiveness loss.

### 4.3. Residual activation behavior

Fig 15 shows the residual activation factors of the proposed FRMRL under Scenario S5. The activation signals are large during the initial transient and then decay as the sliding variables approach the neighborhood of the sliding manifold. The enlarged views show that the activation is not continuously high; instead, it is selectively reintroduced when a low-rate residual condition appears. This behavior is consistent with the design of $\chi_{i}$, which depends jointly on the sliding variable and the physical rate. In other words, the controller does not simply apply a larger gain in all operating regions; it increases the effective reaching effort only when the system has low motion rate while the sliding variable remains nonzero.

**Fig 15:**
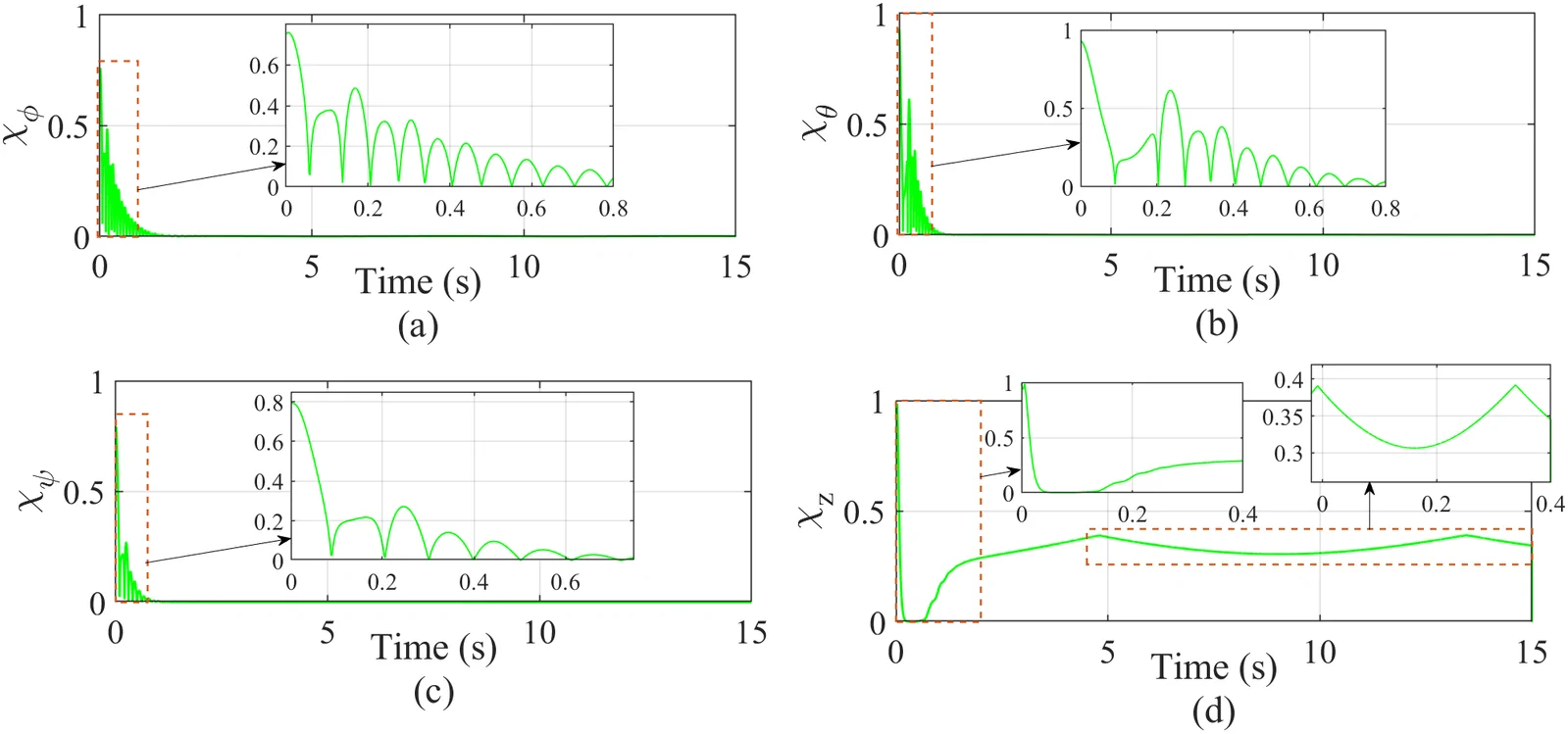
Proposed FRMRL residual activation under Scenario S5: actuator saturation plus dead-zone: (a) roll-channel activation, (b) pitch-channel activation, (c) yaw-channel activation, and (d) altitude-channel activation.

In Scenario S5, the RMS value of the activation factor is 0.1736, indicating that the residual term is sufficiently active to correct dead-zone-induced residual errors, but not large enough to create persistent switching activity. The activation is even stronger under the partial effectiveness-loss case, where RMS($\chi_{i}$)=0.3753, which is consistent with the more severe residual condition after t=6 s. The higher activation in S6 is consistent with the greater residual demand after the specified partial-effectiveness loss; this is a mechanism-level observation for the tested case. The result also supports the theoretical interpretation in Section 3: the proposed term strengthens the Lyapunov descent in the residual regime while preserving smooth control action.

### 4.4. Cross-scenario quantitative assessment

Fig 16 provides a supplementary cross-scenario summary. Panel (b) reports ratios based on the study-specific journal-oriented score defined above; these ratios reflect the selected metrics and weights and are not independent evidence of superiority. The individual tracking and actuator-activity measures reported in Figs 7–15 remain the primary basis for comparing controllers. Panel (c) separately presents the steady-state control-ripple decomposition.

**Fig 16:**
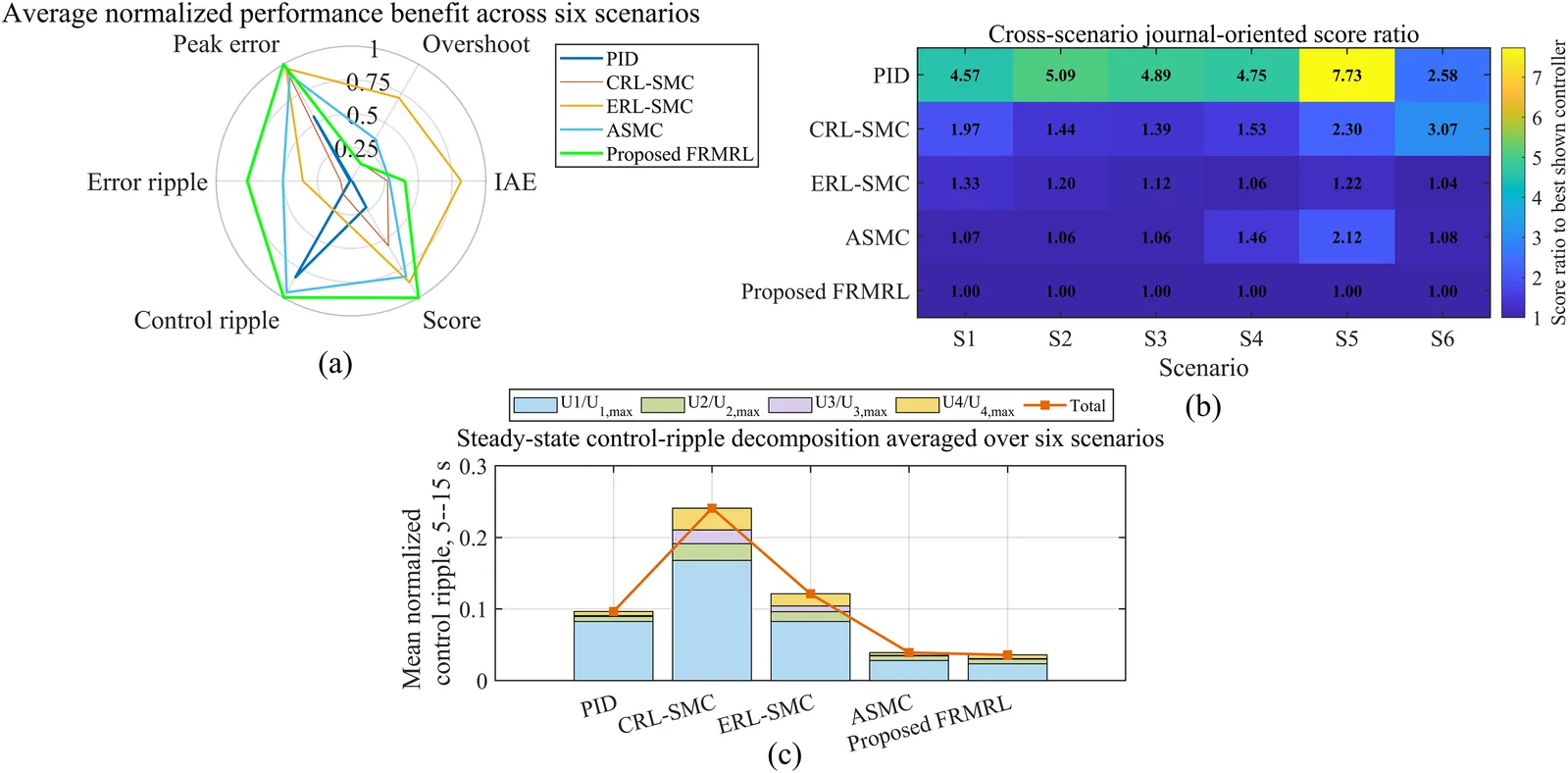
Cross-scenario quantitative summary: (a) average normalized performance benefit across the six scenarios, (b) supplementary ratio of the journal-oriented score relative to FRMRL, and (c) steady-state control-ripple decomposition averaged over the six scenarios.

Across the individual scenarios, FRMRL gives a lower total IAE than ERL-SMC in S1, S5, and S6, whereas ERL-SMC gives a lower total IAE in S2–S4. FRMRL also gives lower control ripple than ERL-SMC in all six scenarios and lower total IAE than SERL-SMC in all six. The mean journal-oriented scores shown in Fig 16 are retained as secondary, weight-dependent summaries: 0.1949 for FRMRL, 0.2245 for ERL-SMC, 0.2427 for ASMC, 0.3892 for CRL-SMC, and 0.9011 for PID. The scenario-level individual metrics, rather than these averages, support the conclusions.

The stacked control-ripple decomposition in Fig 16(c) shows the mean normalized steady-state control ripple across the six scenarios. PID can produce small control variation in benign conditions but loses tracking quality under disturbance and actuator degradation; CRL-SMC provides strong reaching but produces excessive control activity. ERL-SMC yields lower total IAE in S2–S4, whereas FRMRL yields lower total IAE in S1, S5, and S6 and lower control ripple in all six scenarios. These independent metrics show a scenario-dependent trade-off rather than a uniform ranking.

The results lead to three main observations. First, the discontinuous CRL-SMC has strong reaching capability, but its sign-type action introduces excessive torque-command oscillations. This confirms the known limitation of sign-based sliding-mode reaching laws when applied to actuator-limited UAV systems. Second, ERL-SMC yields lower total IAE than FRMRL in S2–S4, whereas FRMRL yields lower total IAE in S1, S5, and S6 and lower control ripple in all six scenarios. These results show a scenario-dependent trade-off between tracking error and actuator activity. Third, the proposed FRMRL improves the smooth-reaching baseline by adding residual corrective effort only in the low-rate residual regime. This explains why the advantage of the proposed method becomes more pronounced in Scenarios S5 and S6, where dead-zone, saturation, motor lag, and partial actuator effectiveness loss generate persistent near-surface residual errors.

The ablation comparison with SERL-SMC is particularly informative. Since SERL-SMC and the proposed FRMRL use identical smooth base gains, the performance difference is caused by the residual term $k_{r}\chi_{i}$, not by a different baseline tuning. Across the six scenarios, the proposed FRMRL reduces the total IAE by 9.16%, 9.86%, 8.22%, 9.27%, 15.97%, and 35.79% relative to SERL-SMC. The largest improvement appears under partial motor effectiveness loss, where the residual mechanism has the strongest activation. This supports the design claim that the proposed term is most useful when the trajectory is close to the sliding surface but cannot eliminate the residual error because the actuator channel has lost part of its effective authority.

Under partial motor-effectiveness loss, FRMRL does not restore the lost actuator authority; it changes when the additional reaching effort is applied. Because the residual term is weighted by both the sliding-variable magnitude and the physical rate, it is attenuated during faster motion and becomes more influential when the rate is low but a residual persists. This differs from the exponential baseline, whose prescribed reaching term remains active throughout the response, and from the tested adaptive baseline, whose gain update depends on the sliding variable but not on this joint rate gate. The lower total IAE and control ripple reported for FRMRL in Scenario S6 are consistent with this mechanism. They support a better balance for the tested partial-loss case, but do not imply superiority when the remaining actuator authority is insufficient to counter the matched perturbation.

FRMRL is not the best method on every individual metric. ERL-SMC gives lower total IAE in S2–S4, while FRMRL gives lower total IAE in S1, S5, and S6. Across all six scenarios, FRMRL yields lower control ripple than ERL-SMC and lower total IAE than SERL-SMC. These results support a scenario-dependent trade-off among tracking error, residual suppression, and actuator smoothness; they do not establish universal superiority.

Across the tested simulations, the FRMRL-based controller provides accurate attitude–altitude tracking and smooth control inputs while reducing actuator-induced residual errors when sufficient control authority remains. The residual activation mechanism is especially useful when the UAV operates near the sliding manifold but the tracking error cannot be fully removed because of dead-zone, saturation, low-frequency perturbations, or partial motor effectiveness loss. These results support the proposed design as a robust and actuator-conscious reaching-law strategy for cascade quadrotor control.

## 5. Conclusion

Actuator-limited quadrotor tracking is governed not only by the nominal robustness of the reaching law, but also by whether the corrective virtual input can actually be delivered by the actuator layer. After the main transient has decayed, saturation, dead-zone behavior, motor lag, rotor drag, and partial effectiveness loss can create a low-rate residual regime: the vehicle motion is small, the sliding variable remains nonzero, and a conventional smooth reaching law may no longer provide sufficient local correction. Discontinuous SMC can force stronger attraction, but it does so by injecting switching activity that is undesirable for finite-bandwidth motors. FRMRL targets this specific regime. Its residual-activated term is not a global gain increase; it is a local smooth correction that becomes influential only when residual sliding motion persists at low angular or vertical rates. Embedded in the cascade attitude–altitude loop, the controller preserves the standard virtual-input implementation while treating actuator nonlinearities as matched channel effects. The Lyapunov analysis supports this interpretation: bounded perturbations lead to uniform ultimate boundedness, the nominal case gives asymptotic convergence, and the residual regime receives a larger local reaching margin than the smooth baseline without restoring discontinuous switching. The scenario-level metrics show a trade-off rather than uniform superiority. ERL-SMC yields lower total IAE than FRMRL in S2–S4, whereas FRMRL yields lower total IAE in S1, S5, and S6 and lower control ripple than ERL-SMC in all six scenarios. FRMRL also reduces total IAE relative to the gain-matched SERL-SMC baseline in every scenario. The composite score in Fig 16 is a secondary, weight-dependent summary and is not a physical measure or the primary basis for these conclusions. The ablation comparison with SERL-SMC is informative because both controllers use the same smooth base gains, but SERL-SMC removes the residual activation. The resulting IAE reductions of 9.16%, 9.86%, 8.22%, 9.27%, 15.97%, and 35.79% across Scenarios S1–S6 show that the improvement is caused by the residual-mode mechanism rather than by more favorable baseline tuning. The largest gain appears under partial motor-effectiveness loss, where the actuator channel has lost usable authority and near-surface residual motion is most difficult to remove. These results position FRMRL as a selective residual-correction mechanism for smooth robust UAV control, rather than as a more aggressive sliding-mode controller. Its practical value lies in improving near-surface error removal while avoiding persistent high-frequency switching. Future work should extend the validation to real-time flight experiments, residual-aware control allocation using measured motor-speed or thrust feedback, and adaptive tuning of the residual-activation parameters under payload variation and progressive rotor-health degradation.
